# A pan-cancer analysis of the oncogenic role of zinc finger protein 419 in human cancer

**DOI:** 10.3389/fonc.2022.1042118

**Published:** 2022-12-12

**Authors:** Weizhen Zhu, Dechao Feng, Xu Shi, Dengxiong Li, Qiang Wei, Lu Yang

**Affiliations:** Department of Urology, Institute of Urology, West China Hospital, Sichuan University, Chengdu, China

**Keywords:** tumor immune microenvironment, pan-cancer, ferroptosis, prognosis, zinc finger protein 419

## Abstract

**Background:**

As a ferroptosis-related gene, the polymorphism of zinc finger protein 419 (ZNF419) at the splice donor site may generate renal cell carcinoma-associated novel minor histocompatibility antigen ZAPHIR. However, the role of ZNF419 in prognosis and immunology in human tumors remains largely unknown. This study aimed to visualize the prognostic landscape of ZNF419 at pan-cancer level and explore the relationship between ZNF419 expression and the tumor immune microenvironment.

**Method:**

Pan-cancer and mutation data were downloaded from TCGA databases and analyzed through R (version 3.6.4) and its suitable packages. Differential ZNF419 expression and prognosis were analyzed. Correlations with ferroptosis-related genes, pathway analysis, tumor stemness, heterogeneity, mutation landscape, and RNA modifications were also explored. The relationships between ZNF419 expression and tumor immunity were investigated through the TIMER and ESTIMATE methods.

**Result:**

ZNF419 was differentially expressed between tumor and normal samples and was associated with overall survival, disease-specific survival and progression-free interval for STES, KIRC, LIHC, LUSC, PRAD, and BLCA. We found the interaction between ZNF419 and FANCD2 might involve in ferroptosis in pan-cancer level. In addition, the mutation frequencies of STES, KIRC, LIHC, LUSC, PRAD, and BLCA were 1.5%, 0.3%, 0.3%, 1.9%, 0.2%, and 0.7%, respectively. We detected that the expression of ZNF419 was closely correlated with most immune checkpoint genes and immune regulatory genes. Furthermore, we found that the ZNF419 expression level was negatively related to the immune score in the six cancers mentioned above. The expression of ZNF419 was significantly associated with various infiltrating immune cells, such as CD4+ T cells, CD8+ T cells, and macrophages in patients with KIRC, PRAD, and LUSC but was only significantly related to macrophages in BLCA patients.

**Conclusion:**

ZNF419 might serve as a potential prognostic and immunological pan-cancer biomarker, especially for KIRC, LIHC, LUSC, PRAD, and BLCA.

## Introduction

To date, cancer is still the leading cause of death and a major problem affecting the patients’ life qualities globally (1). Various cancers, on the other hand, have no absolute cure. As a result, it is critical to investigate more effective treatment strategies, which should include therapy targeting ferroptosis. Ferroptosis is a relatively novel cell death pattern proposed by Dixon in 2012 (2), with distinct characteristics distinguishing it from other types of cell death and the function of recognizing the pathological state of the body. In general, ferroptosis is an iron-dependent programmed cell death caused by lipid peroxidation, which is accompanied by a large amount of iron accumulation, lipid peroxidation, increased reactive oxygen species (ROS), and changes in genes related to iron homeostasis and lipid metabolism (2, 3). Recently, Wang et al. showed that this iron- and lipid ROS-dependent form of programmed cell death might be exploited as a natural and promising therapy for a variety of cancers (4).

Zinc finger protein 419 (ZNF419) is a novel ferroptosis-related gene, whose protein products belong to the human genome’s largest transcription factor family (5). It is meaningful that increasing evidence has shown the underlying roles of zinc finger proteins in cancer progression (5), indicating the potential utility of ZNF419 in cancer research and treatment. Nonetheless, previous research on ZNF419 in tumors has been limited to a few cancer types (6–8). There has yet to be any pan-cancer research into the relationship between ZNF419 expression and multiple cancers. Hence, we used the oncological data for ZNF419 from The Cancer Genome Atlas (TCGA) to determine the prognostic landscape of ZNF419 at the pan-cancer levels. We also explored the possible links between ZNF419 expression and the tumor immune microenvironment and mutation status based on five typical cancers, including lung squamous cell carcinoma (LUSC), bladder urothelial carcinoma (BLCA), kidney renal clear cell carcinoma (KIRC), liver hepatocellular carcinoma (LIHC), and prostate adenocarcinoma (PRAD). Our study shows that ZNF419 can be used as an independent prognostic factor for a variety of cancers and that ZNF419 also plays an important role in tumor immunity. According to our study, ZNF419 is not only a marker for tumor immune microenvironment changes and poor prognosis, but it is also a promising candidate therapeutic target for cancers.

## Methods

### Data acquisition, processing and differential expression analysis

A standardized pan-cancer dataset TCGA Pan-Cancer (PANCAN, N=10535, G=60499) was downloaded from the UCSC database (https://xenabrowser.net/), and expression data of ENSG00000105136 (ZNF419) were extracted in different samples. Then we screened the samples from Solid Tissue Normal, Primary Blood Derived Cancer - Peripheral Blood, and Primary Tumor and filtered samples with 0 expression. Furthermore, log2(x+0.001) transformation was performed for each expression value, while cancer species with fewer than 3 samples were eliminated. We plot these data into a matrix for subsequent analyses. In addition, the differential expression and unpaired Wilcoxon rank sum and signed rank tests were used for differential significance analyses. We also used TIME database to validate the differential expression of ZNF419 in human cancer (9). At protein level, the Human Protein Atlas (HPA) was used to confirmed the differential expression of ZNF419 between tumor and normal samples (10, 11).

### Pan-cancer survival analysis and association with clinical phenotypes

We set overall survival (OS), disease specific survival (DSS), and progression-free interval (PFI) as prognostic indicators. Based on previously extracted data, we screened metastatic samples from the Primary Blood Derived Cancer - Peripheral Blood (TCGA-LAML), Primary Tumor, and TCGA-SKCM databases. Additionally, we integrated the TCGA prognostic database gained from TCGA-related prognostic research in Cell (12). We also eliminated samples with either an expression level of 0 or less than 30 days of follow-up. The complete data matrix was analyzed with the Cox proportional hazards regression model (13) established by the ‘coxph’ function in the ‘survival’ package and the log-rank test was used to obtain prognostic significance. Moreover, we presented the prognosis of selected tumors based on the above results of differential expression of ZNF419 through Kaplan-Meier curves. Unpaired Wilcoxon rank sum and signed rank tests and Kruskal test were utilized to evaluate the correlation between ZNF419 expression and clinical stage and grade. Furthermore, we also explored the relationship between ZNF419 mRNA expression and patient age.

### Correlation of ZNF419 with ferroptosis and pathway analysis

We analyzed the relationship of ZNF419 with twenty-four ferroptosis-related key regulators based on the previous study (14) to better elucidate the role of ZNF419 in ferroptosis. Gene set variation analysis (GSVA) was used to assess the differential pathways between high- and low-expression groups of ZNF419 based on the median value. we calculated the enrichment scores of the related pathways and molecular mechanisms of each sample through R package ‘GSVA’ (15) and ‘c2.cp.v7.4.symbols.gmt’ subset from the molecular signature database (16). The minimum and maximum gene set were 5 and 5000, respectively. Subsequently, ‘wilcox.test’ function was used to evaluate the difference of each pathway between the two clusters. The fold change was 1.3, and we considered p. adj. < 0.01 and false discovery rate < 0.01 as statistical significance.

### Analysis of tumor heterogeneity and stemness

Tumor mutation burden (TMB) reflects the total number of mutations in tumor somatic cells and has been regarded as a marker for evaluating immunotherapy efficacy (17). We downloaded the Simple Nucleotide Variation dataset disposed by MuTect2 software (18) from GDC (https://portal.gdc.cancer.gov/), and applied the ‘tmb’ function in the ‘maftools’ package (version 2.8.05) to calculate the TMB of various cancer species. Mutant-allele tumor heterogeneity (MATH) is another indicator of tumor heterogeneity, and was obtained from the ‘inferHeterogeneity’ function in the ‘maftools’ package (18). Moreover, microsatellite instability (MSI) is also involved in DNA mismatch repair instability (19). Other indicators included neoantigen (NEO), tumor purity, ploidy, homologous recombination deficiency (HRD), and loss of heterozygosity (LOH) (18). Based on somatic mutation data downloaded from TCGA (https://xenabrowser.net/), the relationship between ZNF419 expression and tumor heterogeneity was analyzed *via* using Spearman’s rank correlation coefficient. In addition, we also used six indexes (DNAss, EREG-METHS, DMPss, ENHss, RNAss, and EREG.EXPS) to assess the tumor stemness (20), which was calculated from the characteristics of tumor methylation and mRNA expression.

### Analysis of immunity and the tumor microenvironment

The relationship between ZNF419 expression level and 150 marker genes of 5 types of immune pathways (41 for chemokine, 18 for receptor, 21 for MHC, 24 for immunoinhibitor and 46 for immunostimulator) as well as 60 immune checkpoint genes (inhibitory (24) and stimulatory (36)) obtained from a previous study (21) was included in our analysis. In addition, the Estimation of Stromal and Immune Cells in Malignant Tumor Tissues Using Expression Data (ESTIMATE) algorithm was employed to deduce the degree of either stromal or immune cell infiltration in tumors (22). We employed the ESTIMATE algorithm to calculate the stromal score, immune score, and estimate score in each patient *via* the tumor gene expression profiles, which we extracted and mapped onto GeneSymbol. The Tumor Immune Estimation Resource (TIMER) database, an integrated web server, was also employed for comprehensive analysis of tumor-infiltrating immune cells (9). Therefore, the processed gene expression profiles were further analyzed by the TIMER method in the R package ‘IOBR’ to acquire the immune cell infiltration scores of every tumor sample in each patient, including B cells, CD4+ T cells, CD8+ T cells, neutrophils, macrophages, and dendritic cells (DCs) (23). Moreover, to ensure the accuracy and reliability of the results, the infiltration scores of B cells, cancer-associated fibroblasts (CAFs), CD4+ T cells, CD8+ T cells, macrophages, NK cells, endothelial cells, and other cells were re-evaluated for each patient with the deconvo_epic method in the ‘IOBR’ package (23, 24).

### Relationships between ZNF419 expression and gene mutation and RNA modification

The Simple Nucleotide Variation dataset disposed by MuTect2 software (18) was also used to analyze the gene mutation landscape, and the ‘maftools’ package (version 2.2.10) was used to acquire protein domain information. Moreover, we detected the gene expression and mutation in PRAD, KIRC, LUSC, LIHC, and BLCA by assessing differences in mutation frequencies in each group of samples using chi-square tests. Finally, we used an expression data matrix to analyze the correlations between ZNF419 expression and 44 genes in three classes of RNA modification types, including 10 m1A genes, 13 m5C genes and 21 m6A genes.

### Statistical analysis

Log2(x+0.001) transformation was carried out for each expression value. Two-sided p < 0.05 indicated statistical significance. Wilcoxon rank sum, and signed rank tests were used to analyze pairwise differences, and the Kruskal test was used to test multiple sets of samples. Spearman’s or Pearson’s test was applied to correlation analysis between the two variables used.

## Result

### Differential expression and clinical applications analysis of ZNF419 in pan cancers

**Figure 1:**
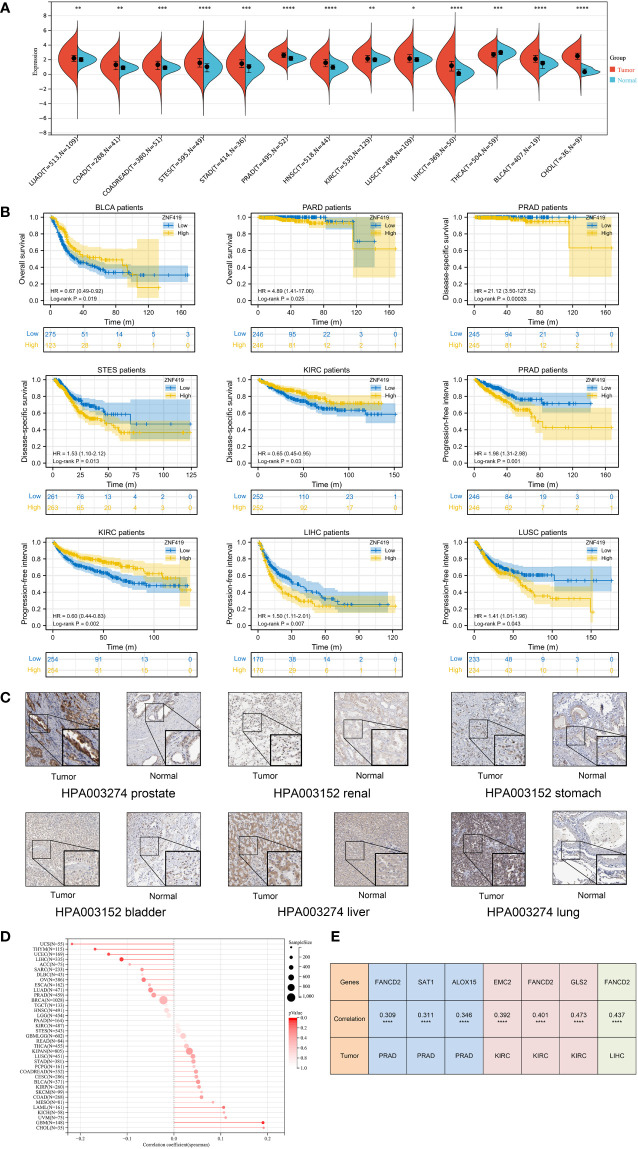
Differential expression analysis, prognosis analysis of ZNF419 in pan-cancer with validation and correlation with ferroptosis-related gene. **(A)** pan-cancer analysis of ZNF419 for differential expression between tumor and normal tissues; **(B)** prognostic analysis with survival curve for BLCA, PRAD, STES, KIRC, LIHC, and LUSC patients; **(C)** validation of protein expression was conducted using HPA database; **(D)** the correlation between patient age and ZNF419 expression; **(E)** correlation analysis of ZNF419 and ferroptosis-related key genes. BLCA, bladder urothelial carcinoma; PRAD, prostate adenocarcinoma; STES, stomach and esophageal carcinoma; KIRC, kidney renal clear cell carcinoma; LIHC, liver hepatocellular carcinoma; LUSC, lung squamous cell carcinoma. "*, **, ***, ****" corresponds to the p value "<0.05, <0.01, <0.001, <0.0001".

To determine the role of ZNF419 in cancer, we evaluated the mRNA expression level of ZNF419 in tumor tissues and adjacent normal tissues. After the calculation and analyses of the data from the UCSC database, we finally obtained the expression data of 26 cancer species. The differential expression of ZNF419 between tumor and normal samples was analyzed (**Supplementary Figure 1**) and we found that ZNF419 was significantly expressed in most cancers, including PRAD, stomach and esophageal carcinoma (STES), KIRC, LUSC, LIHC, and BLCA. Here, we only presented the tumors with statistical significance (**Figure 1A**). The similar results obtained from TIMER differential expression analysis was put in **Supplementary Figure 2**. In addition, to explore the association between ZNF419 expression level and prognosis, we conducted a pan-cancer survival analysis with OS, DSS, and PFI. **Figure 1B** shows the related survival curves. The high expression of ZNF419 was related to lower OS, PFI, or DSS in PRAD, STES, KIRC, LIHC, and LUSC. Cox proportional hazards regression model analysis showed that high ZNF419 expression was significantly correlated with worse OS in glioma (GBMLGG), brain lower grade glioma (LGG), PRAD, and mesothelioma (MESO). However, the situation was reversed in rectal adenocarcinoma (READ) and BLCA, where a low ZNF419 expression level was related to short OS (**Supplementary Figure 3**). Analysis of DSS showed that high ZNF419 expression was associated with poor prognosis in patients with GBMLGG, LGG, cervical squamous cell carcinoma and endocervical adenocarcinoma (CESC), STES, PRAD, MESO, and adrenocortical carcinoma (ACC) (**Supplementary Figure 3**). Nevertheless, in patients with kidney renal papillary cell carcinoma (KIRP), pan-kidney cohort (KIPAN), KIRC, and thymoma (THYM), ZNF419 expression exhibited the opposite relationship with prognosis. In terms of PFI, a similar prognostic value of ZNF419 was found after Cox proportional hazards regression model analysis (**Supplementary Figure 3**). The above results revealed that ZNF419 expression level was an important factor affecting the cancer survival, although their relationships may vary depending on tumor type. Next, we conducted the validation of protein expression using HPA database (**Supplementary Figure 4**) and we found that the ZNF419 protein was expressed higher in tumor tissues than the normal ones in prostate, kidney, stomach, bladder, liver, and lung (**Figure 1C**). We further examined the correlation with clinical phenotypes. We explored the differential expression of ZNF419 according to age for patients with each tumor type and found an age correlation of ZNF419 expression in glioblastoma multiforme (GBM) and LIHC patients (**Figure 1D**). Furthermore, although we also analyzed the association between ZNF419 expression and other clinical phenotypes, including cancer stage, cancer grade and patient gender, the majority of our results were not significant. We put those results in **Supplementary Figure 5**. We further performed correlation analysis of ZNF419 and ferroptosis-related key genes (**Supplementary table 1**). We found the interaction between ZNF419 and FANCD2 might involve in ferroptosis in pan-cancer level, because correlation of ZNF419 and FANCD2 was significant among PRAD, BLCA, KIRC, LIHC, LUSC and STES, especially for PRAD, KIRC and LIHC (**Figure 1E**).

### Relationships of ZNF419 with tumor heterogeneity and stemness

**Figure 2:**
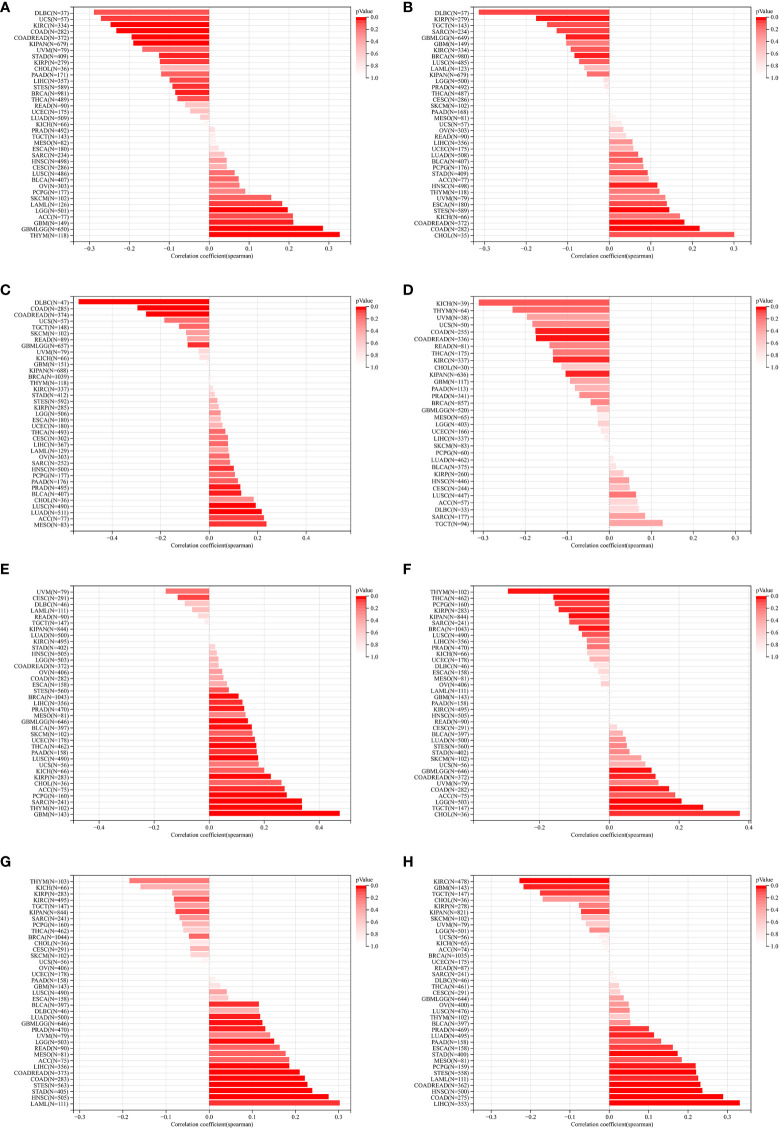
The pan-cancer Spearman analysis of tumor heterogeneity and ZNF419 expression. **(A)** the correlation between TMB and ZNF419 expression level; **(B)** the correlation between MATH and ZNF419 expression level; **(C)** the correlation between MSI and ZNF419 expression level; **(D)** the correlation between NEO and ZNF419 expression level; **(E)** the correlation between tumor purity and ZNF419 expression level; **(F)** the correlation between tumor ploidy and ZNF419 expression level; **(G)** the correlation between HRD and ZNF419 expression level; **(H)** the correlation between LOH and ZNF419 expression level. TMB, tumor mutation burden; MATH, mutant-allele tumor heterogeneity; MSI, microsatellite instability; NEO, neoantigen; HRD, homologous recombination deficiency; LOH, loss of heterozygosity.

**Figure 3:**
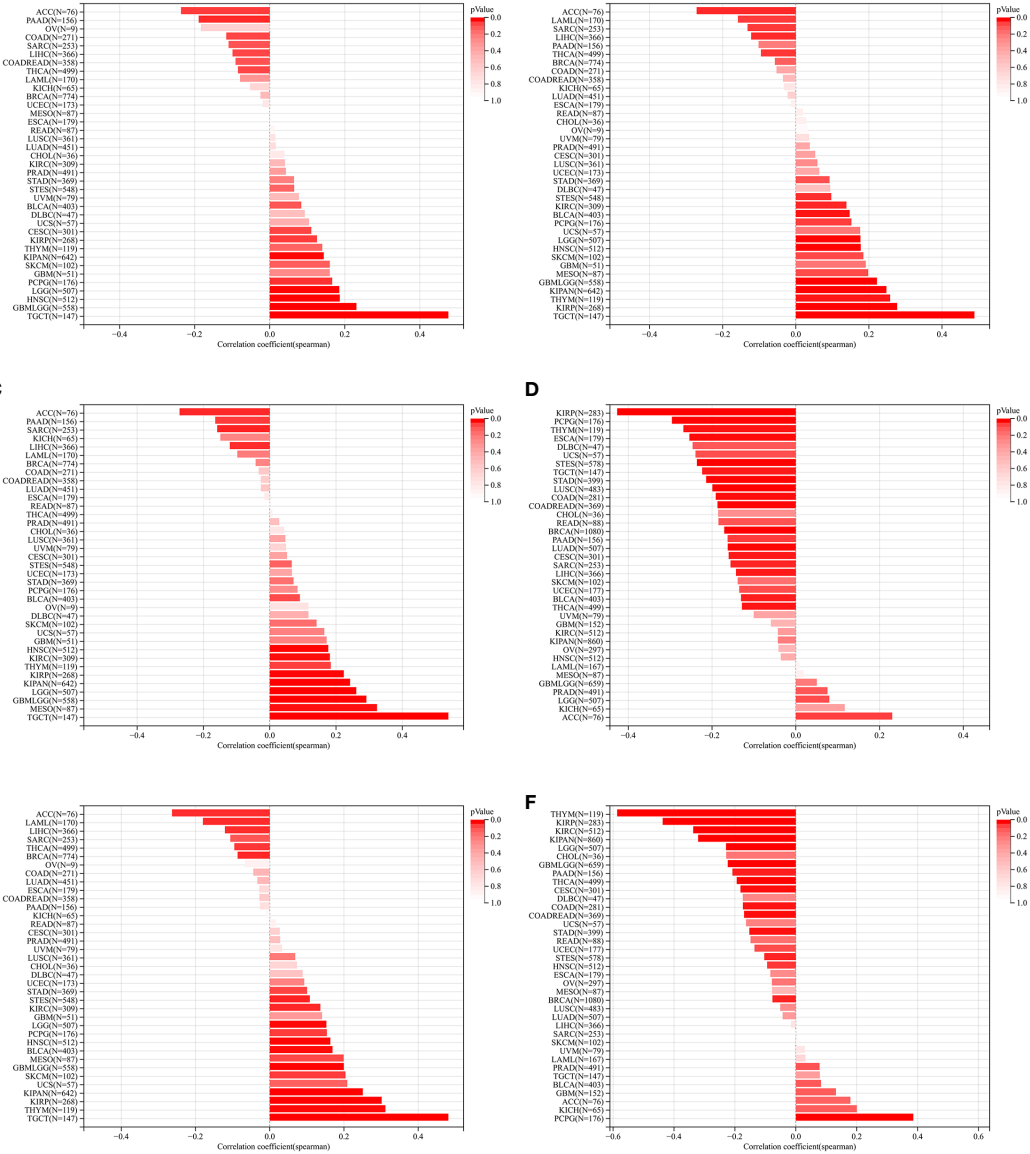
The pan-cancer Spearman analysis of tumor stemness andZNF419 expression. **(A)** the correlation between tumor stemness and ZNF419 expression level using DMPss; **(B)** the correlation between tumor stemness and ZNF419 expression level using DNAss; **(C)** the correlation between tumor stemness and ZNF419 expression level using ENHss; **(D)** the correlation between tumor stemness and ZNF419 expression level using EREG.EXPss; **(E)** the correlatiom between tumor stemness and ZNF419 expression level using EREG-MATHS; **(F)** the correlation between stemness and ZNF419 expression level using RNAss.

We further assessed the relationship between ZNF419 expression and tumor heterogeneity and stemness. Our results demonstrated that the expression level of ZNF419 was significantly related to TMB in 14 cancer species, with 5 being positively related, such as GBM and LGG, and 9 being negatively related, such as KIRC and STES (**Figure 2A**). In terms of MATH, the results showed a positive correlation with the expression of ZNF419 mRNA in 4 types of cancers and a negative correlation in 3 types of cancers (**Figure 2B**). MSI and NEO reflect the response to immunotherapy. Our results demonstrated that in 11 cancers, including PRAD, LUSC, and BLCA, ZNF419 expression was associated with MSI (**Figure 2C**), and that ZNF419 expression was negatively related to NEO in another four cancers (**Figure 2D**). In terms of tumor purity and ploidy, we found a positive correlation between purity and ZNF419 expression in the majority of cancers (**Figure 2E**), but the correlation was not consistent in the tumors where we found a correlation between ploidy and ZNF419 expression, with half showing a positive correlation and the other half showing a negative correlation (**Figure 2F**). HRD status was the key prognostic indicator of multiple tumor therapy strategies and prognosis, as shown in **Figure 2G** in 12 cancers, including STES, LIHC, BLCA, and PRAD. We also observed significant correlations between ZNF419 expression and LOH in various cancers, the majority of which were positive (**Figure 2H**). Moreover, **Figure 3** also depicts the results of the correlation of tumor stemness and ZNF419 expression.

### Relationships between ZNF419 expression and immune regulation and immune infiltration

**Figure 4:**
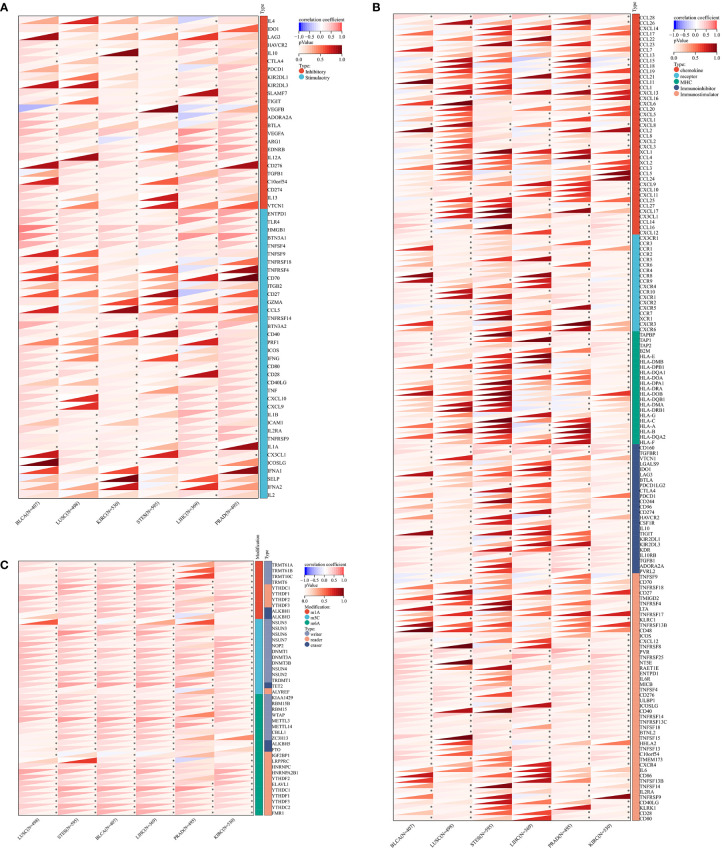
The Spearman analysis of ZNF419 expression and genes functioning in immune checkpoints, immune regulation, and RNA modification. **(A)** the correlation of ZNF419 expression with genes of immune checkpoints; **(B)** the correlation of ZNF419 expression with immune regulatory genes; **(C)** the correlation of ZNF419 expression with genes of RNA modification. *The p value was less than 0.05.

**Figure 5:**
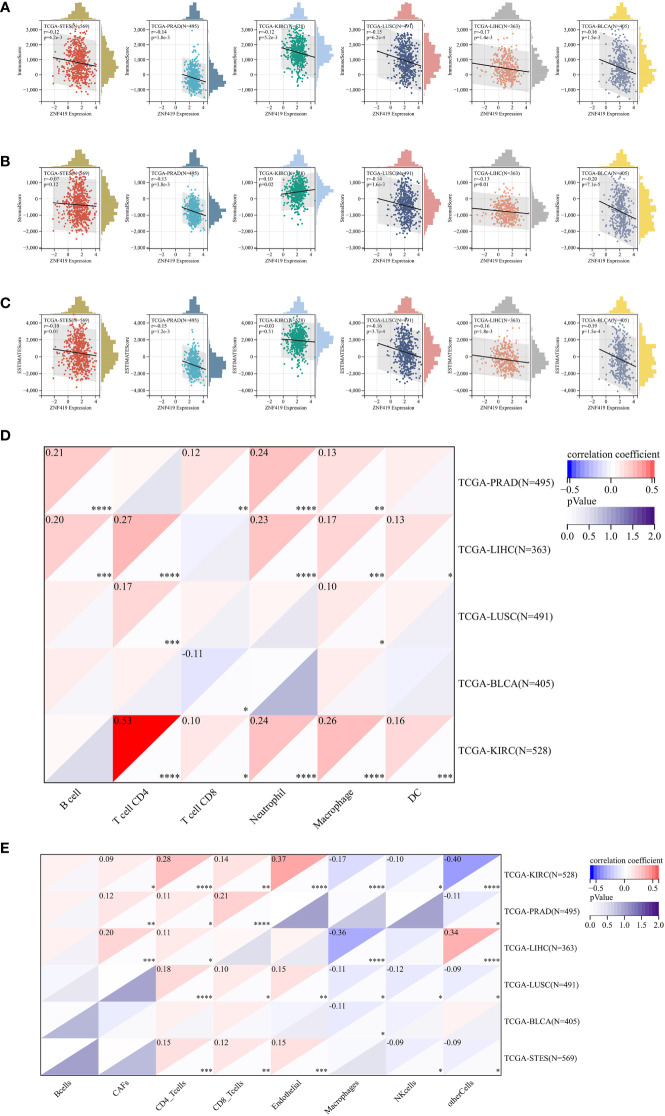
Tumor immune environment and its correlation with ZNF419 expression. **(A)** the correlation of ZNF419 expression with immune score; **(B)** the correlation of ZNF419 expression with stromal score; **(C)** the correlation of ZNF419 expression with estimate score; **(D)** the correlation of ZNF419 expression with immune infiltrating cells using TIMER; **(E)** the correlation of ZNF419 expression with immune infiltrating cells using EPIC. DC, dendritic cells. "*, **, ***, ****" corresponds to the p value "<0.05, <0.01, <0.001, <0.0001".

To illustrate the possible links between ZNF419 expression and immune status in tumors, we conducted an analysis of immune-related genes and immune infiltration conditions in the TME, to explore the role of ZNF419 across cancers from an immune perspective. As a result, **Figures 4A, B** suggests that ZNF419 expression was associated with most immune checkpoint genes and immunoregulatory genes in BLCA, LUSC, KIRC, STES, LIHC, and even in PRAD, which was dubbed the “cold” tumor due to its passive immune profile and poor immunotherapy efficacy. The immune and stromal scores, on the other hand, revealed that ZNF419 expression was negatively correlated with the immune score, stromal score, and ESTIMATE score in most cancer types (**Figures 5A–C**). From the perspective of tumor-infiltrating cells in the TME, the results from TIMER showed that the ZNF491 expression level was significantly positively related to the infiltration of the majority of immune cell types in the TME in PRAD, KIRC, and LIHC (**Figure 5D**). For example, the ZNF419 expression level was positively related to the infiltration of B cells, CD8+ T cells, neutrophils, and macrophages in PRAD, while a positive relationship only existed in CD4+ T cells and macrophages in LUSC (**Figure 5D**). In contrast, ZNF419 expression in BLCA was found to be negatively associated with CD8+ T cells (**Figure 5D**). **Figure 5E** shows partially different results from the previous analysis when using the EPIC method. The findings revealed that there was no link between ZNF419 and B cell infiltration (**Figure 5E**). Furthermore, in KIRC, PRAD, LIHC, LUSC, BLCA, and STES patients, ZNF419 expression was positively associated with CAFs, CD4+ T cells, CD8+ T cells, and endothelial cells but negatively associated with macrophages, NK cells and other cells (**Figure 5E**). Surprisingly, only LIHC patients had a positive correlation between ZNF419 and cell infiltration (**Figure 5E**). Furthermore, ZNF419 expression in BLCA was only related to macrophage infiltration (**Figure 5E**). These partially contradictory findings may point to the importance of epigenetic changes in ZNF419 expression. **Figure 4C** also displayed the results of RNA modification. The expression of ZNF419 was clearly related to the majority of RNA modification genes in LUSC, STES, BLCA, LIHC, PRAD, and KIRC, possibly indicating ZNF419’s important role in epigenetic regulation.

### Relationships between ZNF419 expression and gene mutation, RNA modification, and functional analysis

**Figure 6:**
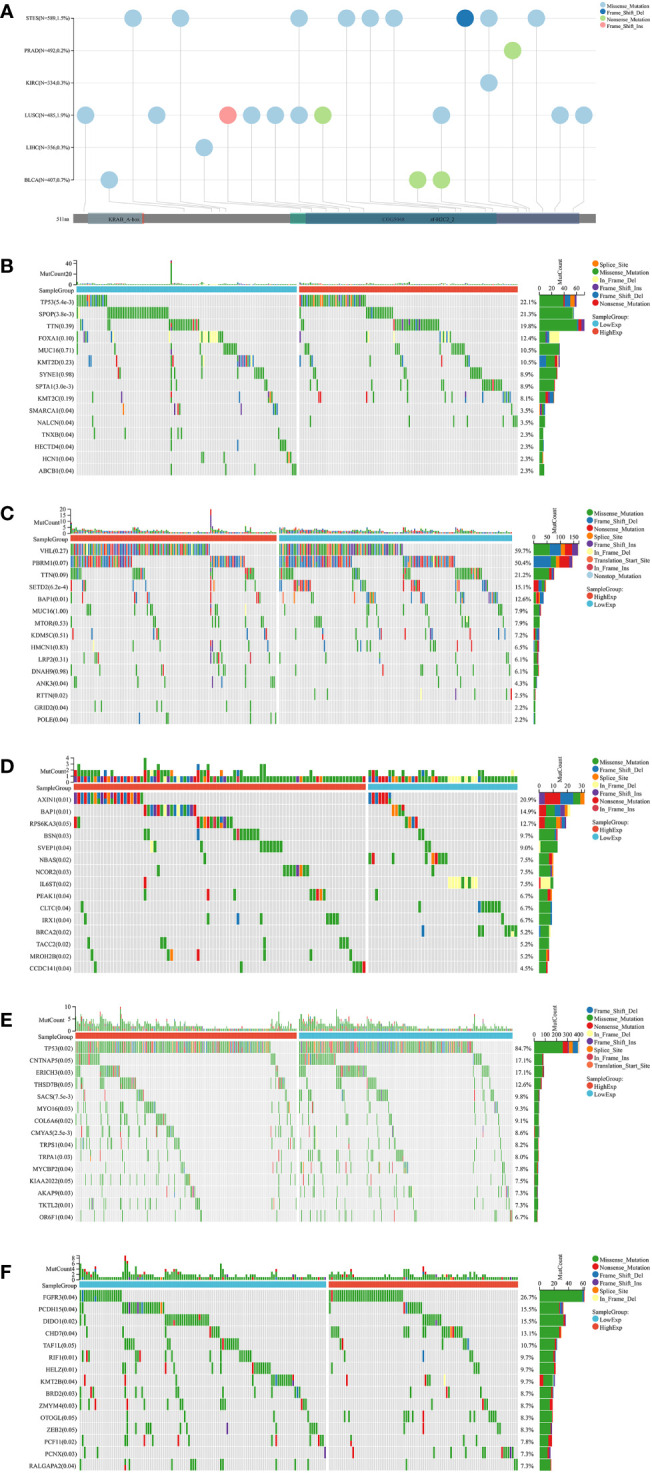
Mutation landscapes analysis of ZNF419. **(A)** mutation landscapes of ZNF419 for STES, PRAD, KIRC, LUSC, LIHC, and BLCA; **(B)** the top 15 mutation genes between high and low-expression of ZNF419 in PRAD patients; **(C)** the top 15 mutation genes between high and low-expression of ZNF419 in KIRC patients; **(D)** the top 15 mutation genes between high and low-expression of ZNF419 in LIHC patients; **(E)** the top 15 mutation genes between high and low-expression of ZNF419 in LUSC patients; **(F)** the top 15 mutation genes between high and low-expression of ZNF419 in BLCA patients. STES, stomach and esophageal carcinoma; PRAD, prostate adenocarcinoma; KIRC, kidney renal clear cell carcinoma; LUSC, lung squamous cell carcinoma; LIHC, liver hepatocellular carcinoma; BLCA, bladder urothelial carcinoma.

**Figure 7:**
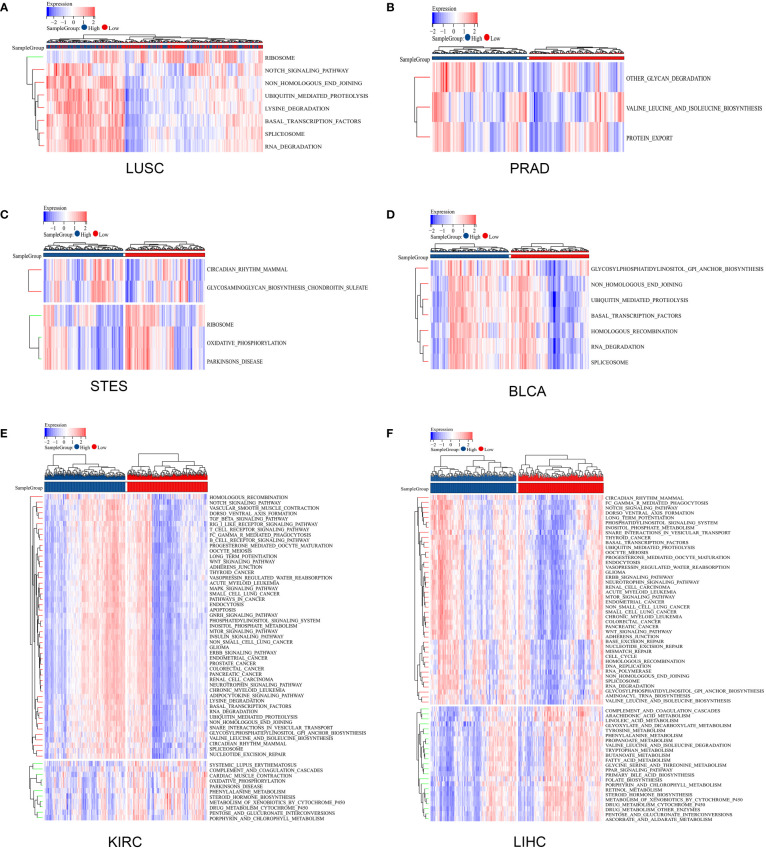
Pathway analysis using gene set variation analysis. **(A)** pathway analysis of ZNF419 in LUSC; **(B)** pathway analysis of ZNF419 in PRAD; **(C)** pathway analysis of ZNF419 in STES; **(D)** pathway analysis of ZNF419 in BLCA; **(E)** pathway analysis of ZNF419 in KIRC; **(F)** pathway analysis of ZNF419 in LIHC. LUSC, lung squamous cell carcinoma; PRAD, prostate adenocarcinoma; STES, stomach and esophageal carcinoma; BLCA, bladder urothelial carcinoma; KIRC, kidney renal clear cell carcinoma; LIHC, liver hepatocellular carcinoma.

Gene mutations in different cancers are closely related to biological functions, clinical phenotypes and therapy responses. The mutation frequencies of KIRC, LIHC, LUSC, PRAD, STES, and BLCA were 0.3%, 0.3%, 1.9%, 0.2%, 1.5%, and 0.7%, respectively (**Figure 6A**). For each cancer, we divided them into two groups according to the expression level of ZNF419, to explore the possible mechanisms and pathways of ZNF419 related to tumorigenesis and cancer progression through their associated mutated genes. In PRAD, TP53, SPOP, and TTN were the top three mutated genes in both the high and low ZNF419 expression groups. However, TNXB, HECTD4, HCN1, and ABCB1 were the genes that mutated in only the low ZNF419 expression group (**Figure 6B**). Similarly, the top three mutated genes in KIRC were VHL, PBRM1 and TTN. Furthermore, mutated RTTN was only studied in the low ZNF419 expression group, whereas mutated GRID2 and POLE were only found in the high expression group (**Figure 6C**). In addition, both groups expressed mutated genes such as AXIN1 and BAP1 in LIHC patients (**Figure 6D**). BRCA2 mutated only in the low ZNF419 expression group, and mutated TACC2, MROH2B, and CCDC141 were found only in the high ZNF419 expression group (**Figure 6D**). In terms of LUSC, the most mutated genes in both groups were TP53 and FGFR3 (**Figure 6E**). The most mutated gene in BLCA patients was FGFR3 (**Figure 6F**). Finally, we conducted pathway analysis of LUSC, PRAD, STES, BLCA, KIRC, and LIHC (**Figure 7**). Overall, the group with high ZNF419 expression level showed more enriched pathways. For example, in LUSC patients, only ribosome-related pathways were enriched in low-ZNF419 expression group, while other pathways including Notch signaling pathway, non-homologous end joining, ubiquitin-mediated proteolysis, basal transcription factors, and spliceosome. In addition, enriched spliceosome-related pathways were also found in STES and BLCA patients with higher expression of ZNF419. As for PRAD, the high-ZNF419 group showed significantly enriched other glycan degradation, valine, leucine, and isoleucine biosynthesis and protein export-related pathways. The enriched pathways in KIRC and LIHC were much more than other four cancers, which may indicate more complex mechanisms of ZNF419 in these two cancers.

## Discussion

Various cancers are still challenging and dangerous diseases, most of which are accompanied by high mortality, poor prognosis and even inevitable death. The occurrence of different cancers not only results in a poor health status for patients but also places a large burden on public health care finance. Selective death of cancer cells induced by multiple therapeutic strategies may act as an efficient way to treat cancers. Remarkably, increasing evidence recently confirms the robust role of ferroptosis in carcinogenesis and cancer therapy (25, 26). Therefore, the expression of the ferroptosis-related gene ZNF419 may account for another novel point in cancer research as well as cancer therapy. However, there have not yet been enough systematic studies on ZNF419 across cancers, with an underlying and poorly illustrated mechanism in the context of ferroptosis.

Our results showed that the expression of ZNF419 was significantly high among 12 cancers. The results of esophageal carcinoma and renal cell carcinoma were consistent with previous studies (6, 7). However, the study conducted by Pils et al. (8) contradicts our current result. They found significantly differential expression of ZNF419 between ovarian cancer tissues and normal control tissues, while we did not, which was possibly attributed to the difference in tumor samples. We also suggested the prognostic value of ZNF419 by analyzing OS, DSS and PFI in different cancers. However, the exact role of ZNF419 in predicting prognosis remains unclear. In our results, high expression of ZNF419 was associated with a good prognosis in some cancers, while in others, low expression of ZNF419 was correlated with a poor prognosis. For example, we found that a high ZNF419 expression level was related to poor DSS in STES patients, which was consistent with a study by Song et al. (7), where they confirmed ZNF419 as a latent hazardous gene. However, ZNF419 seemed to be a protective factor in BLCA, KIRC, PRAD and some other cancers because low ZNF419 expression was related to a good prognosis (analyzed by OS, DSS, and PFI). We hypothesize that this opposite situation may be ascribed to epigenetic regulation, since previous studies have confirmed the alternative splicing polymorphisms of ZNF419 and its related underlying effect in renal cancer (6, 27). Furthermore, the TNM staging system is still a powerful tool with respect to evaluating and predicting the prognosis of multiple cancers, and correspondingly, we analyzed the correlation of ZNF419 expression with TNM staging status. As a result, the significant relationship of ZNF419 with T stage (e.g., BLCA) or M stage (e.g., KIRP) may illuminate the dominant malignant phenotype of ZNF419 in either tumor development or metastasis in different cancer types, possibly resulting from tissue heterogeneity-induced epigenetic regulation. This also suggested that the role of ZNF419 is not exactly the same in different types of cancers, so the therapeutic emphasis is supposed to vary at different cancer types and different cancer stages. In addition, these findings also clearly demonstrate that ZNF419 can be utilized as a biomarker to determine the prognosis of various cancers. However, the TNM staging system also has limitations in elucidating genetic variations, and the powerful heterogeneity present among patients with the same clinical stage may lead to different clinical outcomes. Hence, we conducted the following analyses on genetic and epigenetic regulation as well as immunity to more fully elucidate the role of ZNF419.

Tumor heterogeneity, mediated by genetic (or epigenetic) alterations or caused by evolutionary selection of tumor clones and subclones, leads to molecular biological or genetic changes in tumor progeny cells, ultimately resulting in differences in tumor growth rate, invasiveness, drug sensitivity, prognosis, and other aspects (28, 29). To better elucidate the underlying mechanism and its related regulation patterns of ZNF419, we conducted heterogenic analysis in terms of eight different indexes, such as TMB, MATH, and MSI. As an indicator for tumor heterogeneity, TMB represents the total number of genetic mutations from a molecular perspective indeed. More importantly, as a promising pan-cancer candidate biomarker, the use of TMB guides the selection and management of immunotherapy in the era of precision medicine through predicting immune checkpoint blockade (ICB) response and identifying patients benefiting most, based on the hypothesis that antigenic peptides from increased mutant protein may generate immunogenic new antigens (30, 31). Previous studies have shown the predictive role of TMB in immunotherapy efficacy for non-small-cell lung cancer and colorectal cancer patients (32, 33) and in the prognosis of immunotherapy in pan-cancer patients (34). Our study showed that ZNF419 expression was associated with TMB in 14 cancer species. This may suggest that the level of ZNF419 expression affects tumor heterogeneity at genetic or epigenetic level and changes the TMB of cancers, thereby affecting the patient’s response to ICB therapy. The correlations between ZNF419 expression and MATH in 7 cancer types support our hypothesis. These significant correlations may indicate the potential relationship of ZNF419 expression with mutation and mutation derived heterogeneity due to the characteristic of MATH in reflecting the frequency of all mutant alleles in the tumor by clustering (35). Furthermore, MSI represents the insertion or deletion of repeating units from DNA tracts (36), the high status of which is also suggested to increase the synthesis of several newly formed antigens, thus strengthening the antitumor immune response (37). Our study also demonstrated that ZNF419 expression was correlated with MSI in 11 cancer types. We observed a positive correlation with bladder cancer, and the prognostic significance of MSI for bladder cancer has been confirmed (38). We also found a significant association of ZNF419 expression with PRAD and COAD. Consistent with our results, previous studies have identified that high MSI in colorectal cancer is an independent predictor of clinical characteristics and prognosis (39), and MSI-high PRAD may respond better to ICB (40). Therefore, our analysis of pan-cancer heterogeneity may provide a new reference for predicting the response and prognosis of immunotherapy in different cancers.

Mechanically, we revealed the potential role of ZNF419 in pan-cancer from the perspective of ferroptosis, in which Fanconi anemia complementation group D2 (FANCD2) may serve as a bridge gene along with ZNF419 and ferroptosis. FANCD2 was found to involve in DNA inter-strand crosslinks repair upon stress and inhibit ferroptosis by regulating genes and/or proteins related to iron accumulation and lipid peroxidation (41). In glioblastoma, the expression FANCD2 was confirmed to promote drug resistance through attenuating ferroptosis while the inhibition of FNACD2 increased the ROS level and suppress cell survival (42). We observed the significant correlation of ZNF419 and FANCD2 among PRAD, BLCA, KIRC, LIHC, LUSC and STES, especially for PRAD, KIRC and LIHC. Hence, ZNF419 may regulate ferroptosis through FANCD2 at the pan-cancer level. In addition, our pathway analysis also provided some potential mechanisms. For example, we found the enriched Notch pathways in LUSC patients with high ZNF419 expression level. It is clear that Notch signaling pathway is widely involved in the occurrence and development of malignant tumors. Specifically, it plays a carcinogenic role, leading to dysregulated cell proliferation, cell cycle inhibition, differentiation, and apoptosis, and ultimately results in cell malignant transformation and carcinogenesis (43, 44). Additionally, enriched spliceosome-related pathways in LUSC, BLCA, KIRC, and LIHC may indicate the close relationship between ZNF419 and genetic and epigenetic regulation. It is worth mentioning that the expression of ZNF419 was associated with a variety of pathways in KIRC and LIHC, which may reveal that ferroptosis is not the only mechanism that ZNF419 is involved in the occurrence and development of pan-cancer.

Importantly, our results showed that ZNF419 expression played a vital role in cancer immunity. The persistent interactions between tumor cells and their TME significantly influence tumor initiation, progression, metastasis, and response to therapies (45), and features of the TME could be regarded as markers for assessing the immunotherapy response of tumors (46). In our study, the expression of ZNF419 seemed to play an immunosuppressive role in the TME. According to the ESTIMATE algorithm, significant correlations between ZNF419 expression and the content of both immune and stromal cells in the TME were identified in more than 20 cancer types, and most of them were negative, where PRAD, KIRA, LUSC, LIHC, and BLCA were representative cancer types. It is well known that tumor-infiltrating immune cells may manifest characteristics in both tumor-antagonizing and tumor-promoting functions (47). PRAD is generally considered as one of cold cancers that represent either limited immune cell infiltration or extensive infiltration of immunosuppressive T cells (48). The negative correlation between ZNF419 expression and the infiltration score of immune cells in the TME of PRAD may partly explain this phenomenon because patients with high ZNF419 expression levels had less immune cell infiltration (thus worse antitumor immunity) and worse prognosis. In contrast, although LUSC, BLCA, KIRC and LIHC have been reported to show an inflamed TME and react well to immunotherapy (49–52), the high expression level of ZNF419 still weakened the immune infiltration status in the TME of those cancers and ultimately led to poor prognnosis. To determine the exact alterations in tumor-infiltrating immune cells in the TME under the influence of ZNF419 expression, we assessed the infiltration scores of several different cell types in the TME with the assistance of the TIMER and EPIC methods. ZNF419 expression was closely related to multiple cell types in the TME, and both immune and stromal cells were involved. However, the results were not exactly the same for the two methods. Among several typical cancers with great significance in the above analysis, including KIRC, PRAD, LIHC, LUSC, and BLCA, correlations between B cells and ZNF419 expression were weak or even nonexistent, but associations of ZNF419 expression with T cells were strongly positively related, either CD4+ T cells or CD8+ T cells. Interestingly, a study published in Nature showed that activated T cells, especially CD8+ T cells, enhanced IFN-γ generation and led to lipid peroxidation and subsequent ferroptosis in tumor cells (53). Moreover, increased ferroptosis even enhanced immunotherapy efficacy (53). However, our study contradicted this finding, where high expression level of ZNF419 that predicted a poor prognosis was correlated with more T-cell infiltration. We have a reasonable hypothesis about this. Ferroptosis may play a cancer-promoting role in some specific circumstances. Damage-associated molecular patterns (DAMPs) released during ferroptosis of cancer cells could boost the inflammatory response that supports tumor growth (54, 55). Remarkably, polarization of macrophages to the M2 phenotype mediated by DAMPs during ferroptosis of cancer cells stimulates tumor growth (56), which could also explain some inconsistencies in our own research that correlations of macrophage infiltration from the EPIC and TIMER methods showed opposite results. Of the five cancers mentioned above, the results of method TIMER showed positive correlations of ZNF419 expression and macrophage infiltration, whereas method EPIC showed the opposite, with ZNF419 expression negatively correlated with macrophage infiltration. Apart from the difference in sample sources, the disproportionality of the two types of macrophages may also be a possible reason. The EPIC results may show a negative correlation of ZNF419 expression with M1 macrophages, indicating decreased M1 macrophage infiltration and a suppressive antitumor immune TME under high ZNF419 expression. In contrast, their positive correlations shown in TIMER may suggest more polarization of macrophages to the M2 phenotype and thus the tumor-promoting inflammatory TME. In addition, we found positive correlations between ZNF419 expression and most genes functioning in immune regulation and immune checkpoints, regardless of whether they were immune inhibitory genes or immune stimulatory genes. In summary, our study revealed the unique role of the ferroptotic gene ZNF419 in tumor immunity. Consistent with our results, previous studies revealed the diagnostic and prognostic value of ZNF419 based on its role in immune regulation in epithelial ovarian cancer, esophageal cancer, and renal cancer (6–8). Therefore, the long-term effects of ferroptosis on tumor immunity depend on the interactions between cancer cells and various immune and nonimmune cell subsets in the TME, and the significance of the ZNF419 gene in tumor immunity and pan-cancer immunotherapy should be considered from the perspective of ferroptosis.

To elucidate the role of ZNF419 across cancers from the perspective of gene mutation, we divided patients into two groups according to the ZNF419 expression levels in each type of cancer with relatively high mutation rates (PRAD, KIRC, LIHC, BLCA, and LUSC), and observed similarities and differences in gene mutation conditions between the two groups. Among those five cancer types, we found that TP53 mutation ranked first in both LUSC and PRAD. Mutation of TP53 occurs in approximately 50% among all cancer types, resulting in the loss of wild-type p53 activity and unrestrained tumor progression (57). Remarkably, p53-mediated transcriptional suppression of SLC7A11 contributes to ferroptosis in cancer cells (58), and mutations of TP53 modulate the ability of p53 to promote apoptosis and ferroptosis (59). Regarding the differences between the high- and low-expression groups, we found that TACC2 mutated only in LIHC patients with high ZNF419 expression levels, and high expression of TACC2 in LIHC was associated with poor prognosis (60). In addition, the results also showed that mutated TNXB only existed in the low-expression group of PRAD, while a high expression level of TNXB is correlated with a good survival prognosis in many cancers (61). Similarly, mutated ABCB1 was also found only in the low-expression group of PRAD, and it has been widely identified to take part in drug resistance of PRAD patients (62–64). Not only did ZNF419 employ mutated genes to participate in tumorigenesis and tumor progression, but epigenetic regulation also played an indispensable role. Polymorphism of ZNF419 at the splice donor site may generate renal cell carcinoma-associated novel minor histocompatibility antigen ZAPHIR (6). Supportive evidence was that the expression level of ZNF419 was positively associated with most genes of RNA modification, especially in LUSC, STES, BLCA, LIHC, PRAD, and KIRC, where m1A, m5C and m6A were all involved. Consistent with our results, the prognostic value of m6A RNA modification has been confirmed in LUSC and PRAD patients (65, 66). Moreover, m5C modulators are also independent predictive factors for KIRC patients (67).

However, there were still some limitations in our study even though we searched and integrated data from different databases and conducted subsequent analyses as much as possible. First, bioinformatic analyses did provide insights into the significance of ZNF419 across cancers in terms of cancer immunity, clinical prognosis, and other aspects, but it is still essential to conduct biological validation experiments *in vitro* and *in vivo*. These further experiments are beneficial to elucidate the mechanism of ZNF419 at the molecular and cellular levels and clearly confirm whether ZNF419 expression affects clinical survival through immune and ferroptotic pathways. Acquired results will also be helpful to promote the possible clinical applications of ZNF419 and its related drugs. In addition, our results showed that posttranslational modification may play an important role in the functioning of ZNF419, whereas these databases lack information on posttranslational modification.

In conclusion, the results of this study clarified the close correlations of ZNF419 expression with diverse human cancer types and its related prognostic value. The significant upregulation of ZNF419 in multiple cancers and the negative correlations between the expression of ZNF419 and different cancer species may suggest that ZNF419 can be regarded as an independent prognostic predictive factor for multiple cancers. The different expression levels of ZNF419 in diverse cancers may result in different prognoses, which requires further study. Additionally, our findings indicated the potential mechanisms of ZNF419 expression in tumor heterogeneity, signaling pathways, immunity, and mutations from a pan-cancer perspective. These findings may shed light on the role of ZNF419 in tumorigenesis as well as development and progression across cancers. Moreover, they may enlighten prospective studies focusing on ZNF419 expression and ferroptosis in the immune TME in the future, ultimately providing strategies for immunotherapy with more precision and individuation.

## Conclusions

ZNF419 might serve as a potential prognostic and immunological pan-cancer biomarker, especially for KIRC, LIHC, LUSC, PRAD, and BLCA.

## Data availability statement

The original contributions presented in the study are included in the article/**Supplementary Material**. Further inquiries can be directed to the corresponding authors.

## Author contributions

WZ and DF conducted data analysis, interpreted the data, and drafted the manuscript. WZ, DF, and DL performed the literature search and collected the data. XS and DL contributed to drafting the manuscript and interpreting data. QW and LY supervised the project. All authors reviewed and edited the final manuscript. All authors contributed to the article and approved the submitted version.

## Supplementary material

Supplementary Table 1 Correlation analysis of ZNF419 and ferroptosis-related key genes in renal cancer, stomach cancer, urinary bladder tumor, liver cancer, lung cancer, and prostate cancer.

## References

[1] Sung H Ferlay J Siegel RL Laversanne M Soerjomataram I Jemal A. Global cancer statistics 2020: GLOBOCAN estimates of incidence and mortality worldwide for 36 cancers in 185 countries. CA Cancer J Clin (2021) 71(3):209–49. doi: 10.3322/caac.21660 33538338

[2] Dixon SJ Lemberg KM Lamprecht MR Skouta R Zaitsev EM Gleason CE. Ferroptosis: an iron-dependent form of nonapoptotic cell death. Cell (2012) 149(5):1060–72. doi: 10.1016/j.cell.2012.03.042 PMC3367386 22632970

[3] Dixon SJ. Ferroptosis: bug or feature? Immunol Rev (2017) 277(1):150–7. doi: 10.1111/imr.12533 28462529

[4] Wang H Cheng Y Mao C Liu S Xiao D Huang J. Emerging mechanisms and targeted therapy of ferroptosis in cancer. Mol Ther (2021) 29(7):2185–208. doi: 10.1016/j.ymthe.2021.03.022 PMC8261167 33794363

[5] Jen J Wang YC. Zinc finger proteins in cancer progression. J BioMed Sci (2016) 23(1):53. doi: 10.1186/s12929-016-0269-9 27411336 PMC4944467

[6] Broen K Levenga H Vos J van Bergen K Fredrix H Greupink-Draaisma A. A polymorphism in the splice donor site of ZNF419 results in the novel renal cell carcinoma-associated minor histocompatibility antigen ZAPHIR. PloS One (2011) 6(6):e21699. doi: 10.1371/journal.pone.0021699 21738768 PMC3125305

[7] Song J Liu Y Guan X Zhang X Yu W Li Q. A novel ferroptosis-related biomarker signature to predict overall survival of esophageal squamous cell carcinoma. Front Mol Biosci (2021) 8:675193. doi: 10.3389/fmolb.2021.675193 34291083 PMC8287967

[8] Pils D Tong D Hager G Obermayr E Aust S Heinze G. A combined blood based gene expression and plasma protein abundance signature for diagnosis of epithelial ovarian cancer–a study of the OVCAD consortium. BMC Cancer (2013) 13:178. doi: 10.1186/1471-2407-13-178 23551967 PMC3639192

[9] Li T Fan J Wang B Traugh N Chen Q Liu JS. TIMER: A web server for comprehensive analysis of tumor-infiltrating immune cells. Cancer Res (2017) 77(21):e108–10. doi: 10.1158/1538-7445.AM2017-108 PMC6042652 29092952

[10] Uhlen M Zhang C Lee S Sjöstedt E Fagerberg L Bidkhori G. A pathology atlas of the human cancer transcriptome. Science (2017) 357(6352):eaan2507. doi: 10.1126/science.aan2507 28818916

[11] Uhlén M Fagerberg L Hallström BM Lindskog C Oksvold P Mardinoglu A. Proteomics. tissue-based map of the human proteome. Science (2015) 347(6220):1260419. 25613900 10.1126/science.1260419

[12] Liu J Lichtenberg T Hoadley KA Poisson LM Lazar AJ Cherniack AD. An integrated TCGA pan-cancer clinical data resource to drive high-quality survival outcome analytics. Cell (2018) 173(2):400–16.e11. doi: 10.1016/j.cell.2018.02.052 29625055 PMC6066282

[13] Andersen PK Gill RD. Cox's regression model for counting processes: A Large sample study. J Ann Stat (1982) 10(4):1100–20. doi: 10.1214/aos/1176345976

[14] Feng D Shi X Zhu W Zhang F Li D Han P. A pan-cancer analysis of the oncogenic role of leucine zipper protein 2 in human cancer. Exp Hematol Oncol (2022) 11(1):55. doi: 10.1186/s40164-022-00313-x 36109820 PMC9476580

[15] Hänzelmann S Castelo R Guinney J. GSVA: gene set variation analysis for microarray and RNA-seq data. BMC Bioinf (2013) 14:7. doi: 10.1186/1471-2105-14-7 PMC3618321 23323831

[16] Liberzon A Subramanian A Pinchback R Thorvaldsdóttir H Tamayo P Mesirov JP. Molecular signatures database (MSigDB) 3.0. Bioinformatics (2011) 27:1739–40 doi: 10.1093/bioinformatics/btr260 PMC3106198 21546393

[17] Choucair K Morand S Stanbery L Edelman G Dworkin L Nemunaitis J. TMB: a promising immune-response biomarker, and potential spearhead in advancing targeted therapy trials. Cancer Gene Ther (2020) 27(12):841–53. doi: 10.1038/s41417-020-0174-y 32341410

[18] Beroukhim R Mermel CH Porter D Wei G Raychaudhuri S Donovan J. The landscape of somatic copy-number alteration across human cancers. Nature (2010) 463(7283):899–905. doi: 10.1038/nature08822 20164920 PMC2826709

[19] Vilar E Gruber SB. Microsatellite instability in colorectal cancer-the stable evidence. Nat Rev Clin Oncol (2010) 7(3):153–62. doi: 10.1038/nrclinonc.2009.237 PMC3427139 20142816

[20] Malta TM Sokolov A Gentles AJ Burzykowski T Poisson L Weinstein JN. Machine learning identifies stemness features associated with oncogenic dedifferentiation. Cell (2018) 173(2):338–54.e15. doi: 10.1016/j.cell.2018.03.034 29625051 PMC5902191

[21] Thorsson V Gibbs DL Brown SD Wolf D Bortone DS Ou Yang TH. The immune landscape of cancer. Immunity (2018) 48(4):812–30. doi: 10.1016/j.immuni.2018.03.023 PMC5982584 29628290

[22] Yoshihara K Shahmoradgoli M Martínez E Vegesna R Kim H Torres-Garcia W. Inferring tumour purity and stromal and immune cell admixture from expression data. Nat Commun (2013) 4:2612. doi: 10.1038/ncomms3612 24113773 PMC3826632

[23] Zeng D Ye Z Shen R Yu G Wu J Xiong Y. IOBR: Multi-omics immuno-oncology biological research to decode tumor microenvironment and signatures. Front Immunol (2021) 12:687975. doi: 10.3389/fimmu.2021.687975 34276676 PMC8283787

[24] Racle J de Jonge K Baumgaertner P Speiser DE Gfeller D. Simultaneous enumeration of cancer and immune cell types from bulk tumor gene expression data. Elife (2017) 6:e26476. doi: 10.7554/eLife.26476 29130882 PMC5718706

[25] Mou Y Wang J Wu J He D Zhang C Duan C. Ferroptosis, a new form of cell death: opportunities and challenges in cancer. J Hematol Oncol (2019) 12(1):34. doi: 10.1186/s13045-019-0720-y 30925886 PMC6441206

[26] Zhao L Zhou X Xie F Zhang L Yan H Huang J. Ferroptosis in cancer and cancer immunotherapy. Cancer Commun (Lond) (2022) 42(2):88–116. doi: 10.1002/cac2.12250 35133083 PMC8822596

[27] Martin G Selcuklu SD Schouest K Nembaware V McKeown PC Seoighe C. Allele-specific splicing effects on DKKL1 and ZNF419 transcripts in HeLa cells. Gene (2017) 598:107–12. doi: 10.1016/j.gene.2016.11.004 27826023

[28] McGranahan N Swanton C. Clonal heterogeneity and tumor evolution: Past, present, and the future. Cell (2017) 168(4):613–28. doi: 10.1016/j.cell.2017.01.018 28187284

[29] Pe'er D Ogawa S Elhanani O Keren L Oliver T G Wedge D. Tumor heterogeneity. Cancer Cell (2021) 39(8):1015–7. doi: 10.1016/j.ccell.2021.07.009 34375606

[30] Chan TA Yarchoan M Jaffee E Swanton C Quezada SA Stenzinger A. Development of tumor mutation burden as an immunotherapy biomarker: utility for the oncology clinic. Ann Oncol (2019) 30(1):44–56. doi: 10.1093/annonc/mdy495 30395155 PMC6336005

[31] Gubin MM Artyomov MN Mardis ER Schreiber RD. Tumor neoantigens: building a framework for personalized cancer immunotherapy. J Clin Invest. (2015) 125(9):3413–21. doi: 10.1172/JCI80008 PMC4588307 26258412

[32] Devarakonda S Rotolo F Tsao MS Lanc I Brambilla E Masood A. Tumor mutation burden as a biomarker in resected non-Small-Cell lung cancer. J Clin Oncol (2018) 36(30):2995–3006. doi: 10.1200/JCO.2018.78.1963 30106638 PMC6804865

[33] Lee DW Han SW Bae JM Jang H Han H Kim H. Tumor mutation burden and prognosis in patients with colorectal cancer treated with adjuvant fluoropyrimidine and oxaliplatin. Clin Cancer Res (2019) 25(20):6141–7. doi: 10.1158/1078-0432.CCR-19-1105 31285374

[34] Samstein RM Lee CH Shoushtari AN Hellmann MD Shen R Janjigian YY. Tumor mutational load predicts survival after immunotherapy across multiple cancer types. Nat Genet (2019) 51(2):202–6. doi: 10.1038/s41588-018-0312-8 PMC6365097 30643254

[35] Mroz EA Rocco JW. MATH, a novel measure of intratumor genetic heterogeneity, is high in poor-outcome classes of head and neck squamous cell carcinoma. Oral Oncol (2013) 49(3):211–5. doi: 10.1016/j.oraloncology.2012.09.007 PMC3570658 23079694

[36] Hause RJ Pritchard CC Shendure J Salipante SJ. Classification and characterization of microsatellite instability across 18 cancer types. Nat Med (2016) 22(11):1342–50. doi: 10.1038/nm.4191 27694933

[37] Zhao P Li L Jiang X Li Q. Mismatch repair deficiency/microsatellite instability-high as a predictor for anti-PD-1/PD-L1 immunotherapy efficacy. J Hematol Oncol (2019) 12(1):54. doi: 10.1186/s13045-019-0738-1 31151482 PMC6544911

[38] Tural D Akar E Baytekin HF Canoglu D Yilmaz M Tugcu V. Relationship between survival outcomes and microsatellite instability, tumor infiltrating lymphocytes and programmed cell death ligand-1 expression in patients with bladder cancer and radical cystectomy. J buon. (2021) 26(5):2117–25. 34761625

[39] Gryfe R Kim H Hsieh ET Aronson MD Holowaty EJ Bull SB. Tumor microsatellite instability and clinical outcome in young patients with colorectal cancer. N Engl J Med (2000) 342(2):69–77. doi: 10.1056/NEJM200001133420201 10631274

[40] Le DT Uram JN Wang H Bartlett BR Kemberling H Eyring AD. PD-1 blockade in tumors with mismatch-repair deficiency. N Engl J Med (2015) 372(26):2509–20. doi: 10.1056/NEJMoa1500596 PMC4481136 26028255

[41] Song X Xie Y Kang R Hou W Sun X Epperly MW. FANCD2 protects against bone marrow injury from ferroptosis. Biochem Biophys Res Commun (2016) 480(3):443–9. doi: 10.1016/j.bbrc.2016.10.068 PMC6591579 27773819

[42] Song L Wu J Fu H Wu C Tong X Zhang M. Abnormally expressed ferroptosis-associated FANCD2 in mediating the temozolomide resistance and immune response in glioblastoma. Front Pharmacol (2022) 13:921963. doi: 10.3389/fphar.2022.921963 35754466 PMC9213730

[43] Meurette O Mehlen P. Notch signaling in the tumor microenvironment. Cancer Cell (2018) 34(4):536–48. doi: 10.1016/j.ccell.2018.07.009 30146333

[44] Marignol L Rivera-Figueroa K Lynch T Hollywood D. Hypoxia, notch signalling, and prostate cancer. Nat Rev Urol. (2013) 10(7):405–13. doi: 10.1038/nrurol.2013.110 PMC5240418 23712204

[45] Xiao Y Yu D. Tumor microenvironment as a therapeutic target in cancer. Pharmacol Ther (2021) 221:107753. doi: 10.1016/j.pharmthera.2020.107753 33259885 PMC8084948

[46] Wu T Dai Y. Tumor microenvironment and therapeutic response. Cancer Lett (2017) 387:61–8. doi: 10.1016/j.canlet.2016.01.043 26845449

[47] Lei X Lei Y Li JK Du WX Li RG Yang J. Immune cells within the tumor microenvironment: Biological functions and roles in cancer immunotherapy. Cancer Lett (2020) 470:126–33. doi: 10.1016/j.canlet.2019.11.009 31730903

[48] Majidpoor J Mortezaee K. The efficacy of PD-1/PD-L1 blockade in cold cancers and future perspectives. Clin Immunol (2021) 226:108707. doi: 10.1016/j.clim.2021.108707 33662590

[49] Middleton G Brock K Savage J Mant R Summers Y Connibear J. Pembrolizumab in patients with non-small-cell lung cancer of performance status 2 (PePS2): a single arm, phase 2 trial. Lancet Respir Med (2020) 8(9):895–904. doi: 10.1016/S2213-2600(20)30033-3 32199466

[50] Necchi A Anichini A Raggi D Briganti A Massa S Lucianò R. Pembrolizumab as neoadjuvant therapy before radical cystectomy in patients with muscle-invasive urothelial bladder carcinoma (PURE-01): An open-label, single-arm, phase II study. J Clin Oncol (2018) 36(34):3353–60. doi: 10.1200/JCO.18.01148 30343614

[51] Choueiri TK Tomczak P Park SH Venugopal B Ferguson T Chang YH. Adjuvant pembrolizumab after nephrectomy in renal-cell carcinoma. N Engl J Med (2021) 385(8):683–94. doi: 10.1056/NEJMoa2106391 34407342

[52] Sangro B Sarobe P Hervás-Stubbs S Melero I. Advances in immunotherapy for hepatocellular carcinoma. Nat Rev Gastroenterol Hepatol (2021) 18(8):525–43. doi: 10.1038/s41575-021-00438-0 PMC8042636 33850328

[53] Wang W Green M Choi JE Gijón M Kennedy PD Johnson JK. CD8(+) T cells regulate tumour ferroptosis during cancer immunotherapy. Nature (2019) 569(7755):270–4. doi: 10.1038/s41586-019-1170-y PMC6533917 31043744

[54] Tang D Kang R Coyne CB Zeh HJ Lotze MT. PAMPs and DAMPs: signal 0s that spur autophagy and immunity. Immunol Rev (2012) 249(1):158–75. doi: 10.1111/j.1600-065X.2012.01146.x PMC3662247 22889221

[55] Wen Q Liu J Kang R Zhou B Tang D. The release and activity of HMGB1 in ferroptosis. Biochem Biophys Res Commun (2019) 510(2):278–83. doi: 10.1016/j.bbrc.2019.01.090 30686534

[56] Dai E Han L Liu J Xie Y Kroemer G Klionsky DJ. Autophagy-dependent ferroptosis drives tumor-associated macrophage polarization *via* release and uptake of oncogenic KRAS protein. Autophagy (2020) 16(11):2069–83. doi: 10.1080/15548627.2020.1714209 PMC7595620 31920150

[57] Bykov VJN Eriksson SE Bianchi J Wiman KG. Targeting mutant p53 for efficient cancer therapy. Nat Rev Cancer (2018) 18(2):89–102. doi: 10.1038/nrc.2017.109 29242642

[58] Jiang L Kon N Li T Wang SJ Su T Hibshoosh H. Ferroptosis as a p53-mediated activity during tumour suppression. Nature (2015) 520(7545):57–62. doi: 10.1038/nature14344 25799988 PMC4455927

[59] Chen X Kang R Kroemer G Tang D. Broadening horizons: the role of ferroptosis in cancer. Nat Rev Clin Oncol (2021) 18(5):280–96. doi: 10.1038/s41571-020-00462-0 33514910

[60] Shakya M Zhou A Dai D Zhong Q Zhou Z Zhang Y. High expression of TACC2 in hepatocellular carcinoma is associated with poor prognosis. Cancer biomark (2018) 22(4):611–9. doi: 10.3233/CBM-170091 PMC6130418 29843208

[61] Liot S Aubert A Hervieu V Kholti NE Schalkwijk J Verrier B. Loss of tenascin-X expression during tumor progression: A new pan-cancer marker. Matrix Biol Plus. (2020) 6-7:100021. doi: 10.1016/j.mbplus.2020.100021 33543019 PMC7852205

[62] Lombard AP Liu C Armstrong CM D'Abronzo LS Lou W Chen H. Overexpressed ABCB1 induces olaparib-taxane cross-resistance in advanced prostate cancer. Transl Oncol (2019) 12(7):871–8. doi: 10.1016/j.tranon.2019.04.007 PMC6510951 31075528

[63] Lombard AP Lou W Armstrong CM D'Abronzo LS Ning S Evans CP. Activation of the ABCB1 amplicon in docetaxel- and cabazitaxel-resistant prostate cancer cells. Mol Cancer Ther (2021) 20(10):2061–70. doi: 10.1158/1535-7163.MCT-20-0983 PMC8492550 34326198

[64] Seo HK Lee SJ Kwon WA Jeong KC. Docetaxel-resistant prostate cancer cells become sensitive to gemcitabine due to the upregulation of ABCB1. Prostate (2020) 80(6):453–62. doi: 10.1002/pros.23946 32134535

[65] Li N Zhan X. Identification of pathology-specific regulators of m(6)A RNA modification to optimize lung cancer management in the context of predictive, preventive, and personalized medicine. Epma J (2020) 11(3):485–504. doi: 10.1007/s13167-020-00220-3 32849929 PMC7429590

[66] Quan Y Zhang X Ping H. Construction of a risk prediction model using m6A RNA methylation regulators in prostate cancer: comprehensive bioinformatic analysis and histological validation. Cancer Cell Int (2022) 22(1):33. doi: 10.1186/s12935-021-02438-1 35045837 PMC8772220

[67] Wu J Hou C Wang Y Wang Z Li P Wang Z. Comprehensive analysis of m(5)C RNA methylation regulator genes in clear cell renal cell carcinoma. Int J Genomics (2021) 2021:3803724. doi: 10.1155/2021/3803724 34631874 PMC8497170

