# Supplementary material for: A pan-cancer analysis of the oncogenic role of zinc finger protein 419 in human cancer
Source: Front Oncol. 2022 Dec 12;12:1042118. doi: 10.3389/fonc.2022.1042118 (PMC9791222; doi:10.3389/fonc.2022.1042118)
Supplement: Supplementary Table 1 — Correlation analysis of ZNF419 and ferroptosis-related key genes in renal cancer, stomach cancer, urinary bladder tumor, liver cancer, lung cancer, and prostate cancer. [file DataSheet_1.docx]

**Supplementary figure 1.** The differential expression of ZNF419 in human cancer.


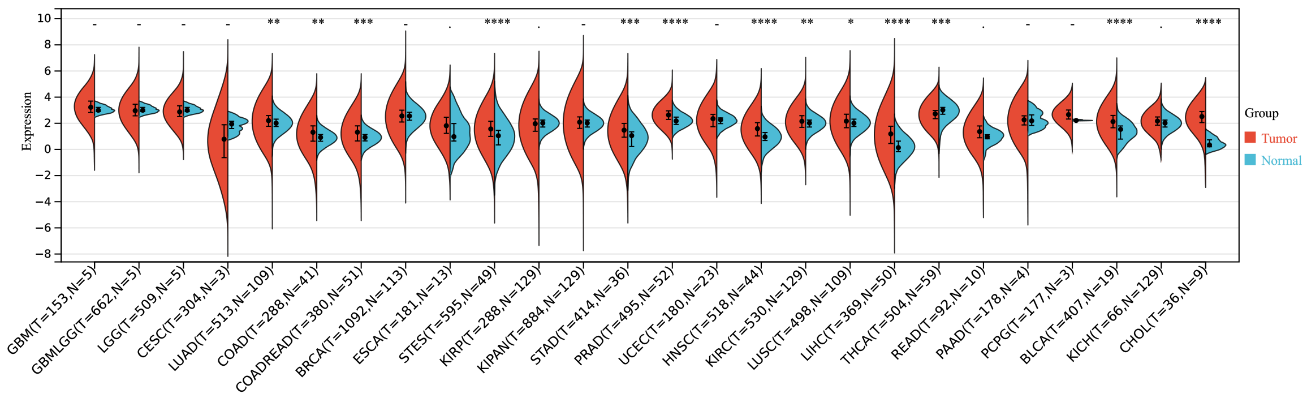


**Supplementary figure 2.** The differential expression of ZNF419 in human cancer using TIMER database.


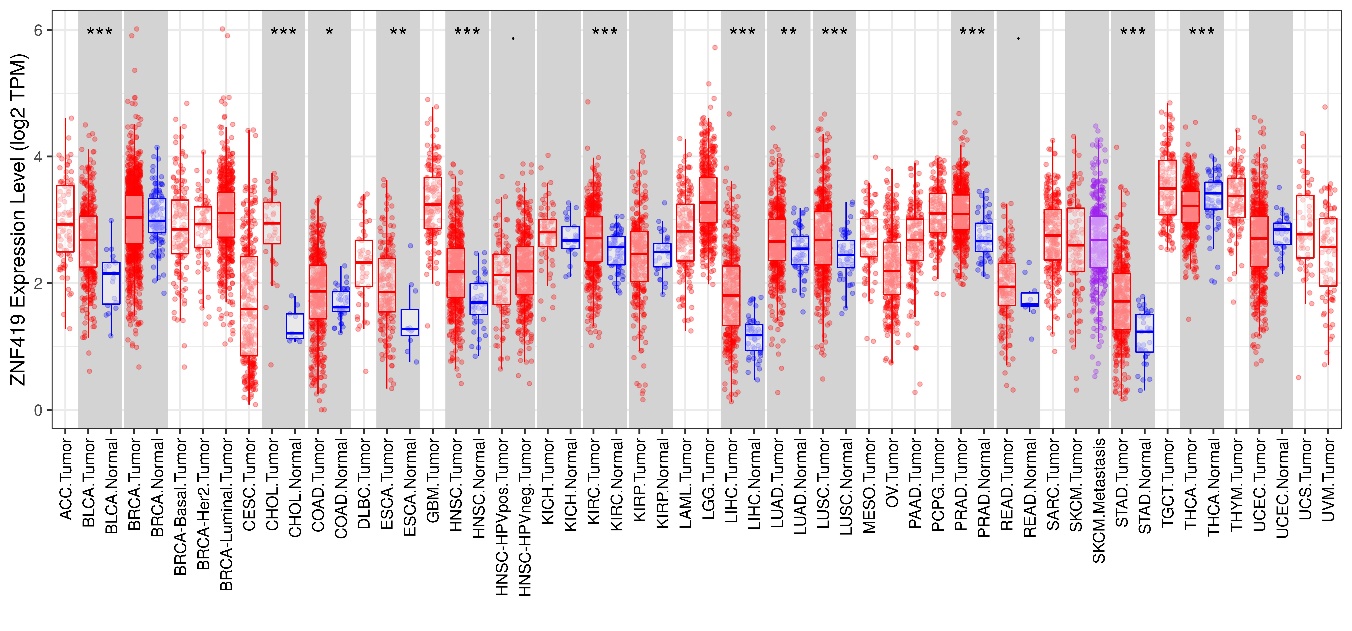


**Supplementary figure 3.** The prognosis effect of ZNF419 on human cancer.

Overall survival


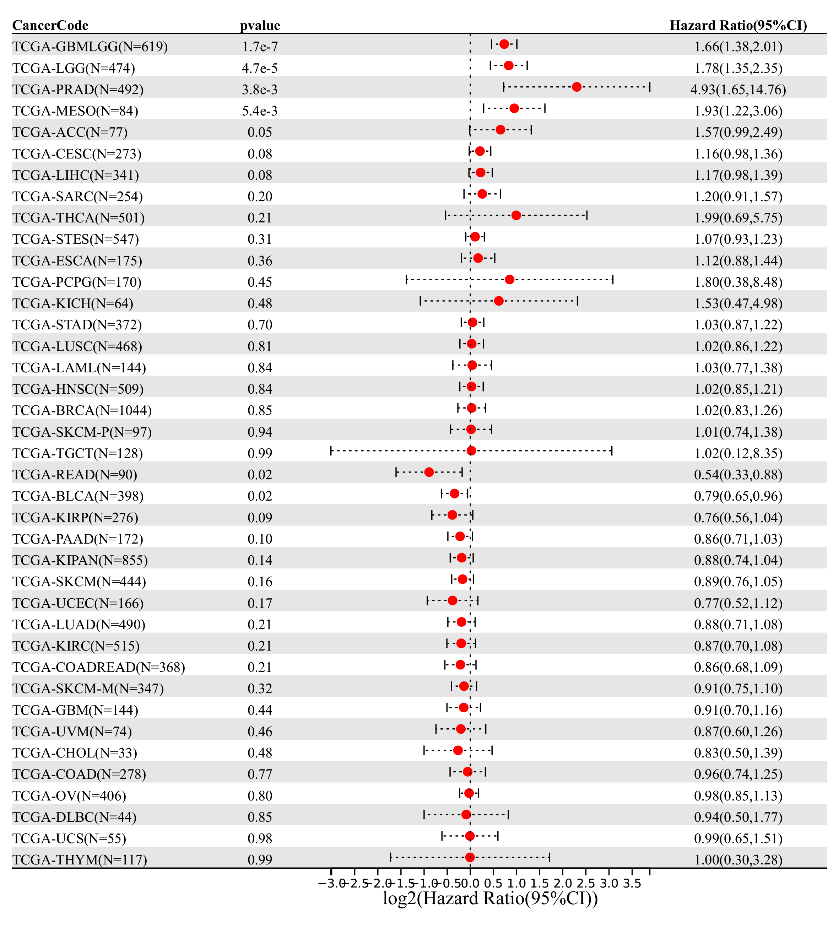


Disease-specific survival


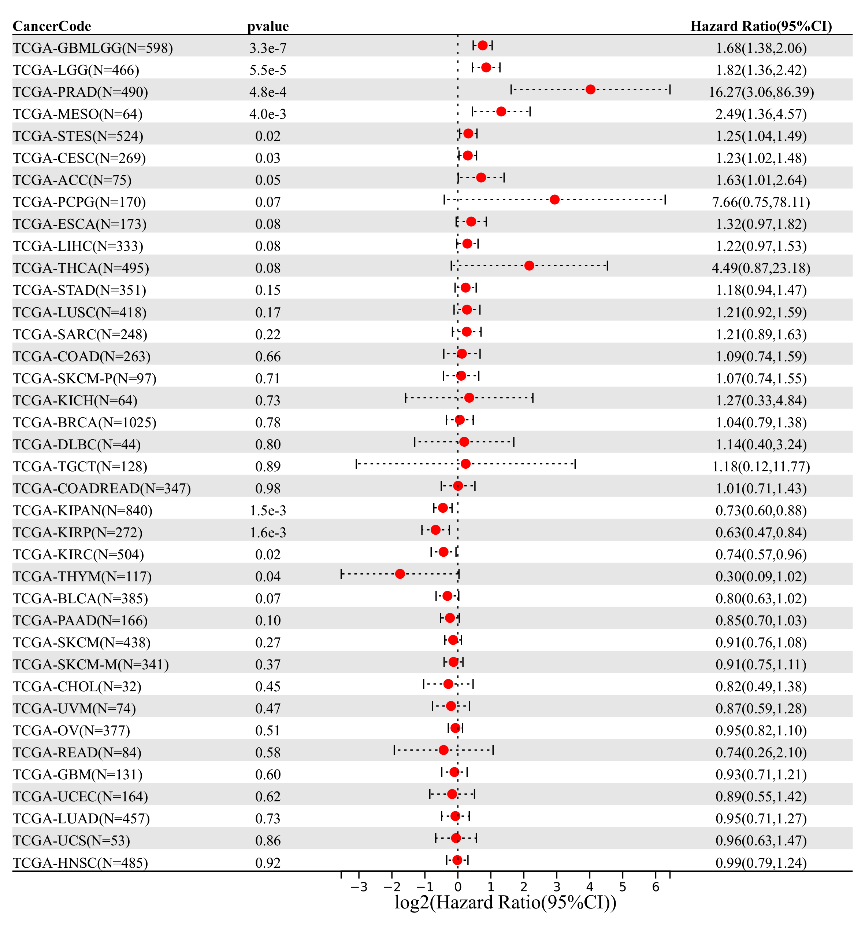


Progression-free interval


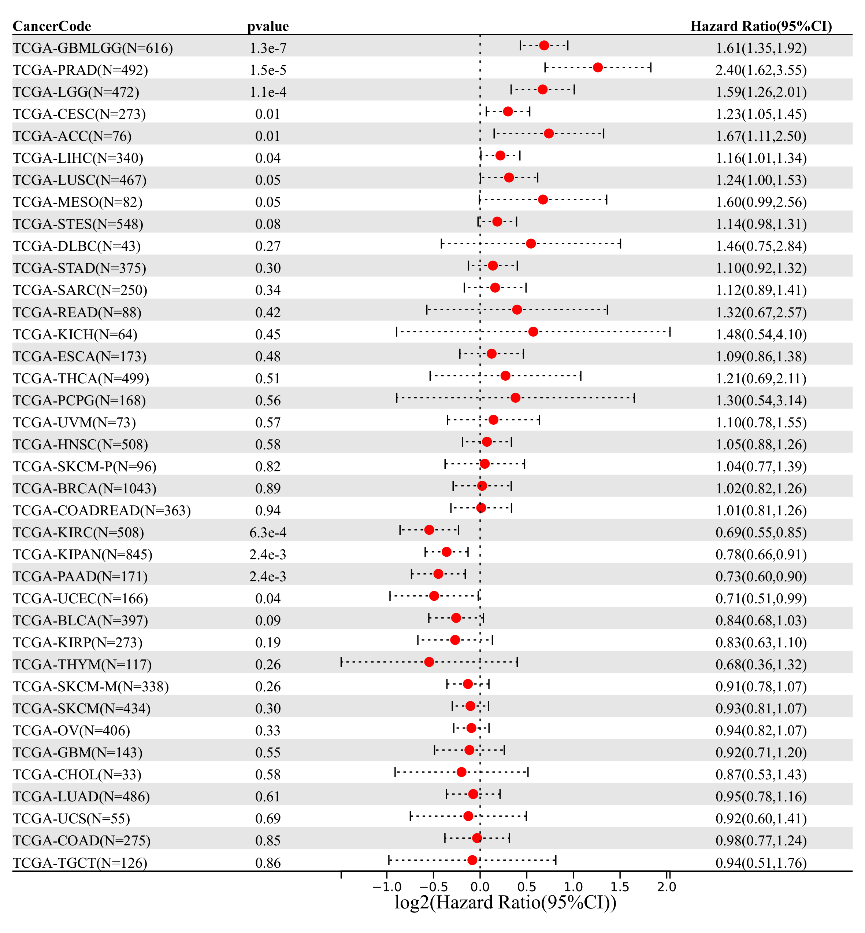


**Supplementary figure 4.** The differential expression of ZNF419 between tumor and normal samples across PRAD, KIRC, BLCA, LIHC, LUSC and STAD using HPA database.

HPA003152 renal cancer medium


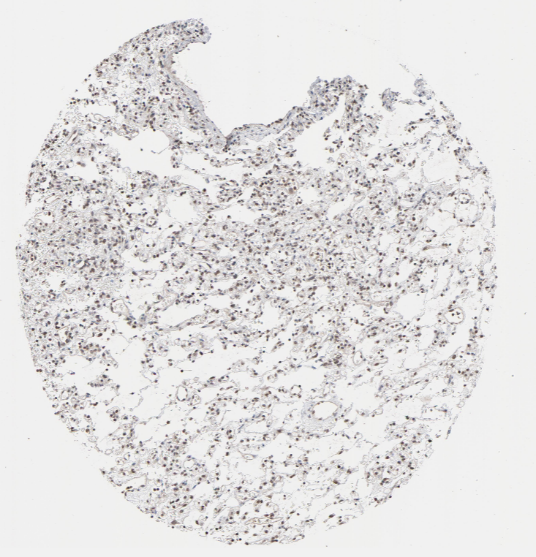


HPA003152 renal normal not detected


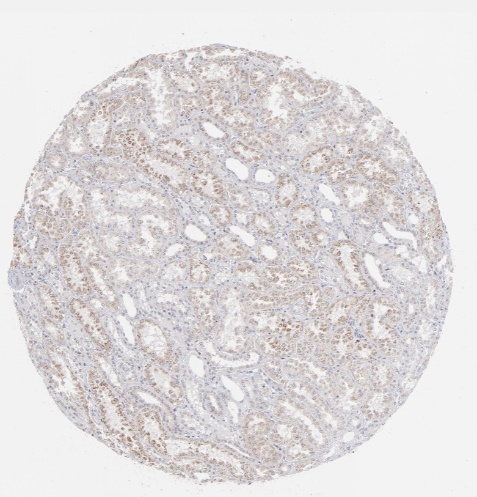


HPA003152 stomach cancer high


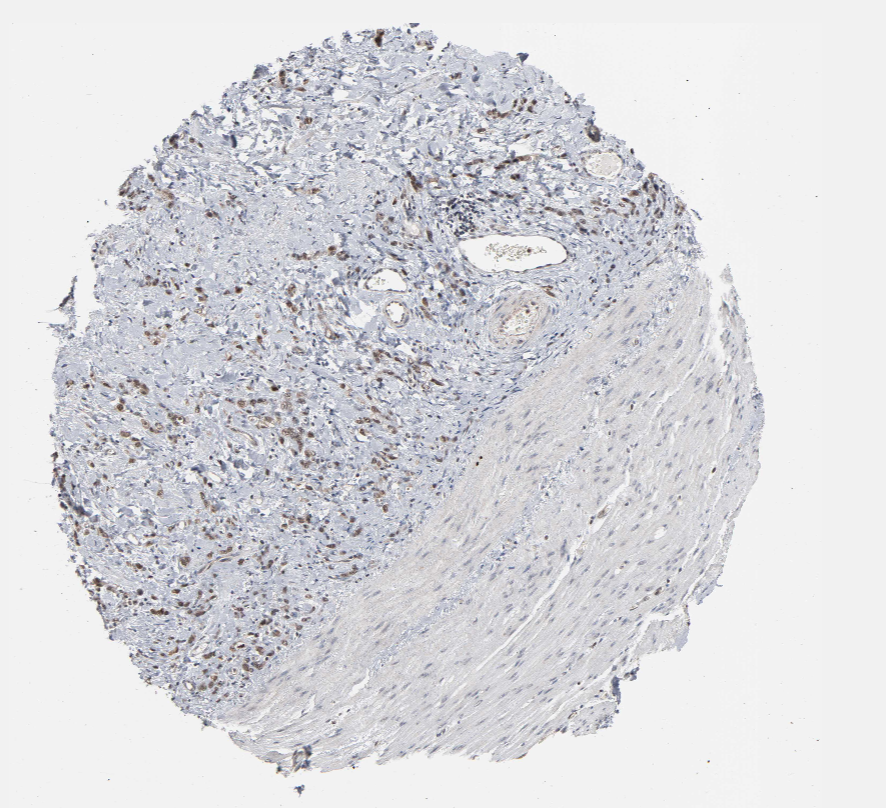


HPA003152 stomach normal medium


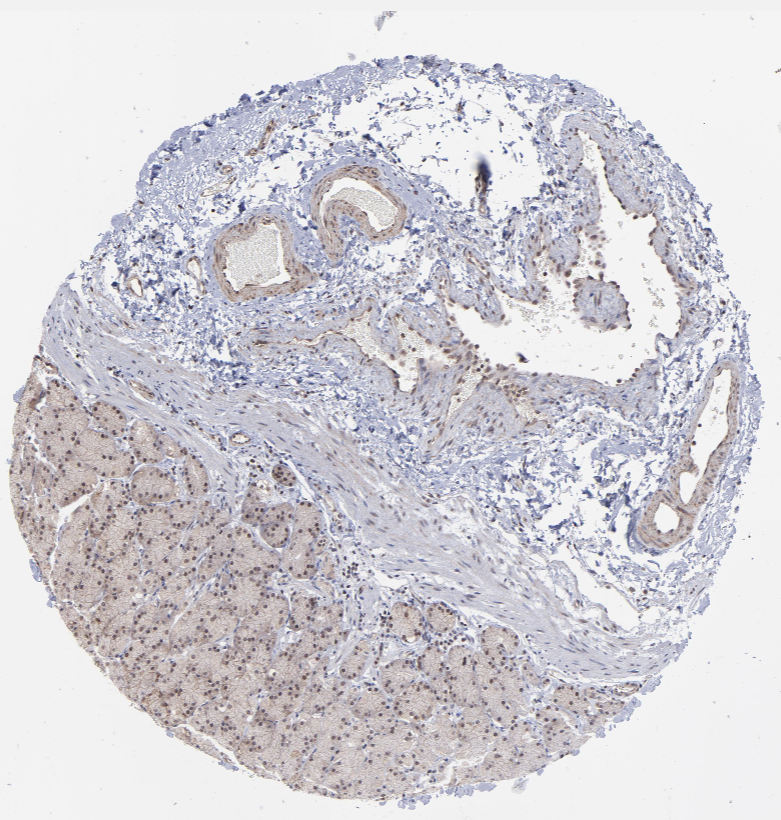


HPA003152 urinary bladder normal medium


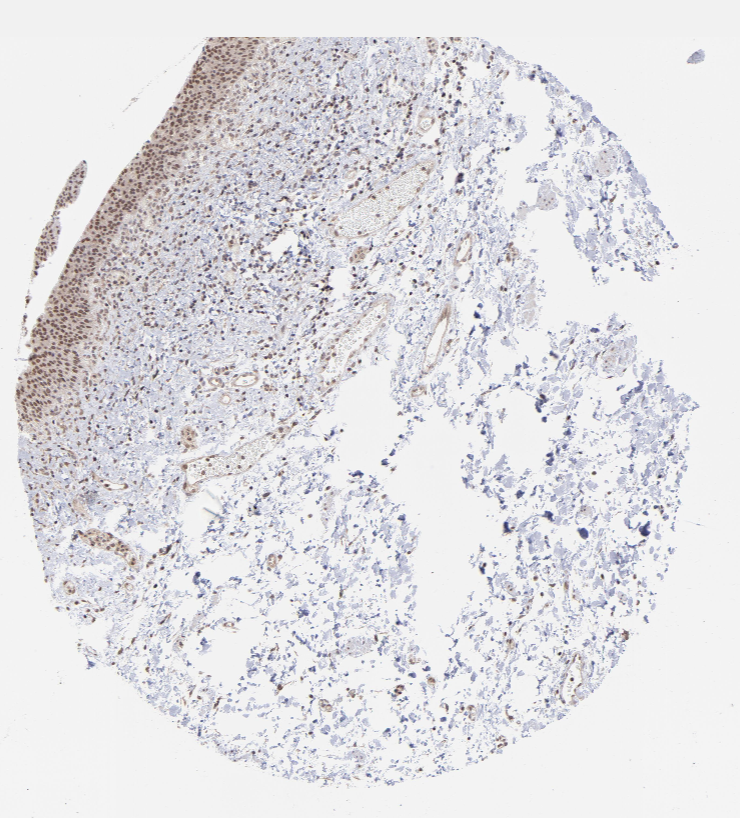


HPA003152 urinary bladder tumor high


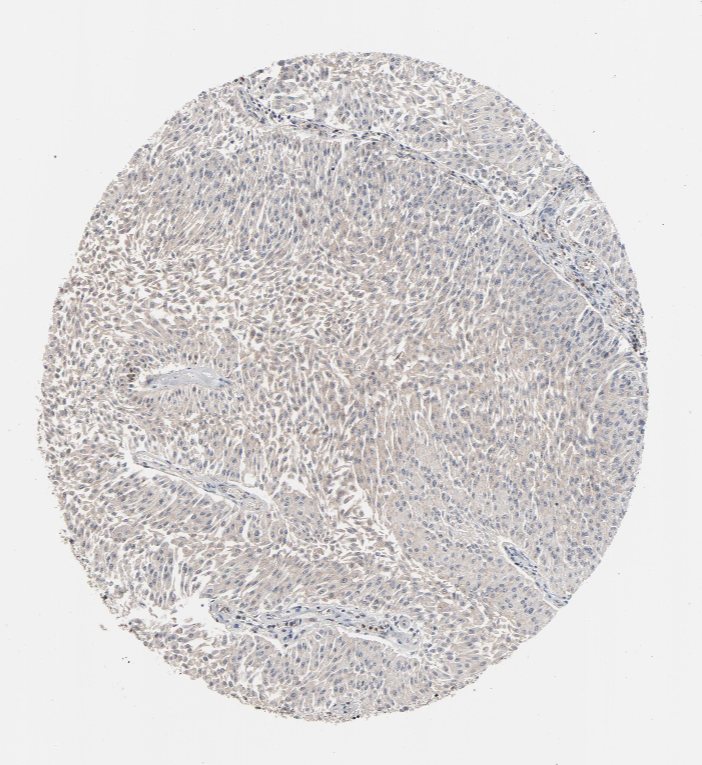


HPA003274 liver cancer medium


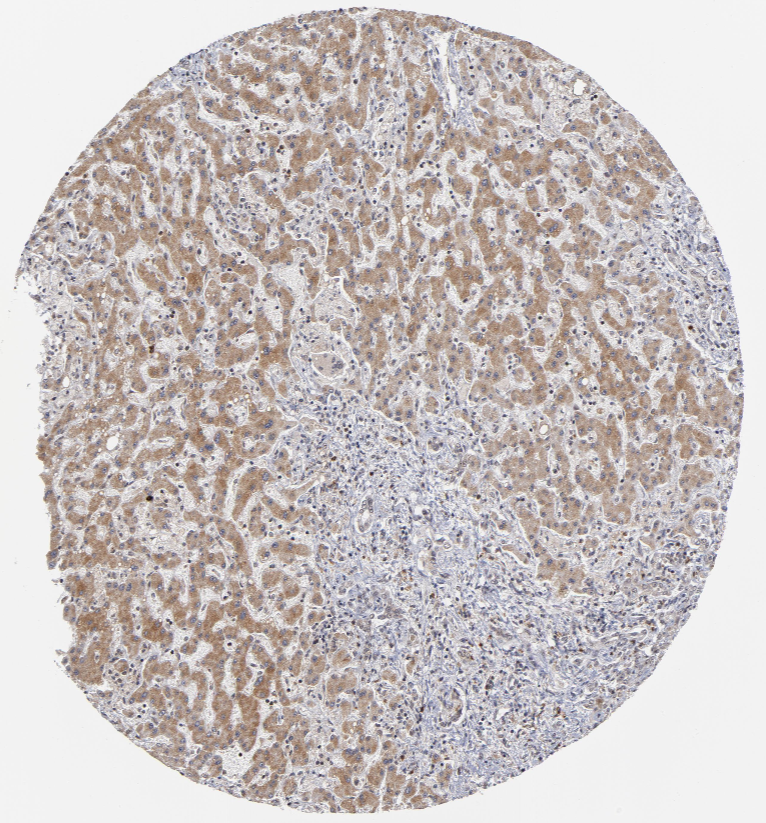


HPA003274 liver normal not detected


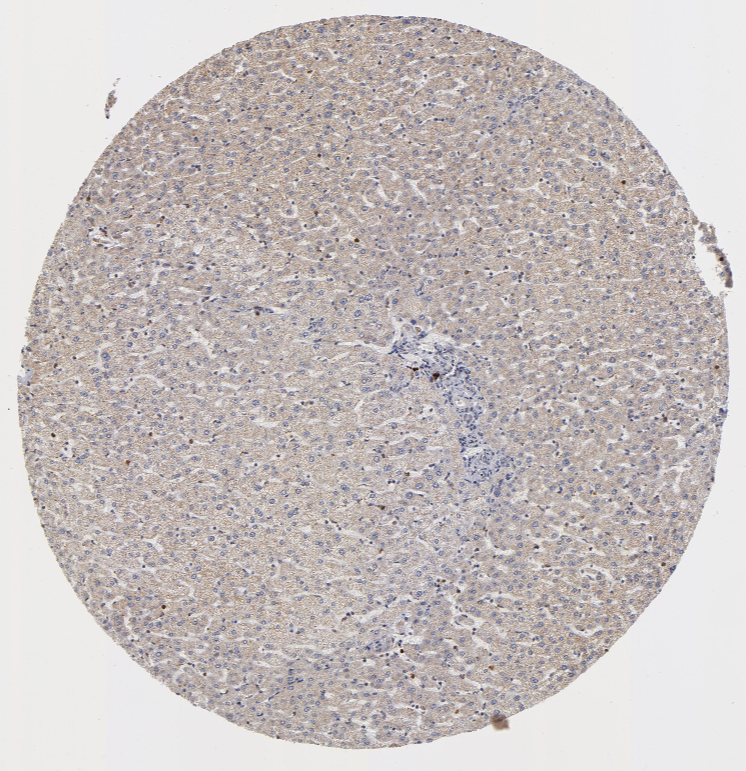


HPA003274 lung cancer medium


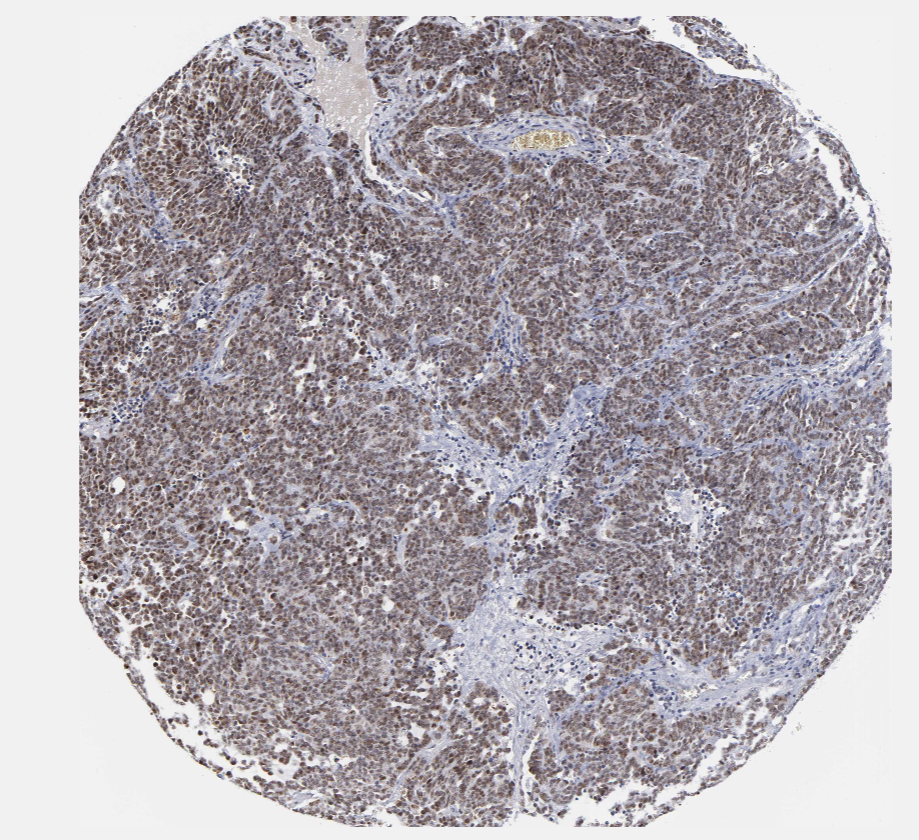


HPA003274 lung normal not detected


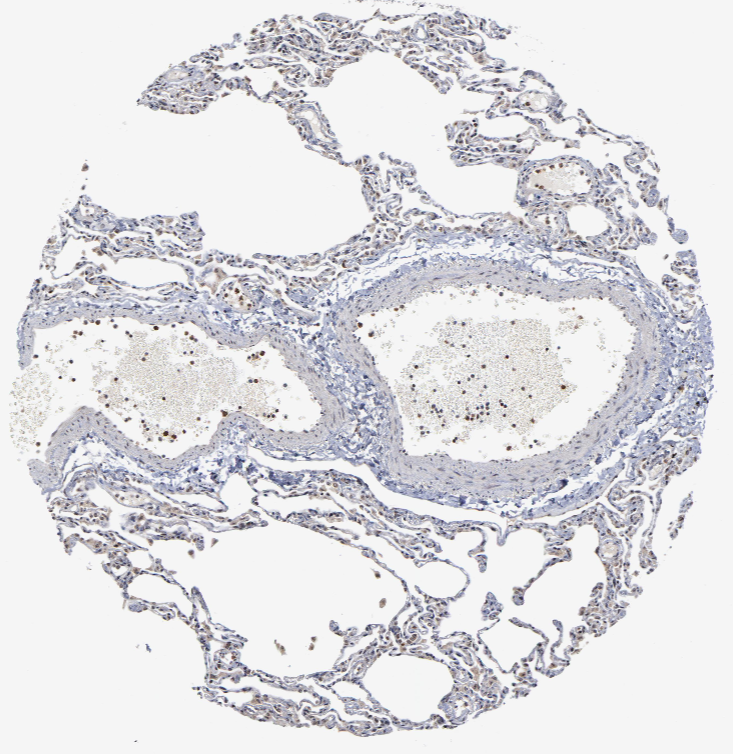


HPA003274 prostate cancer tumor high


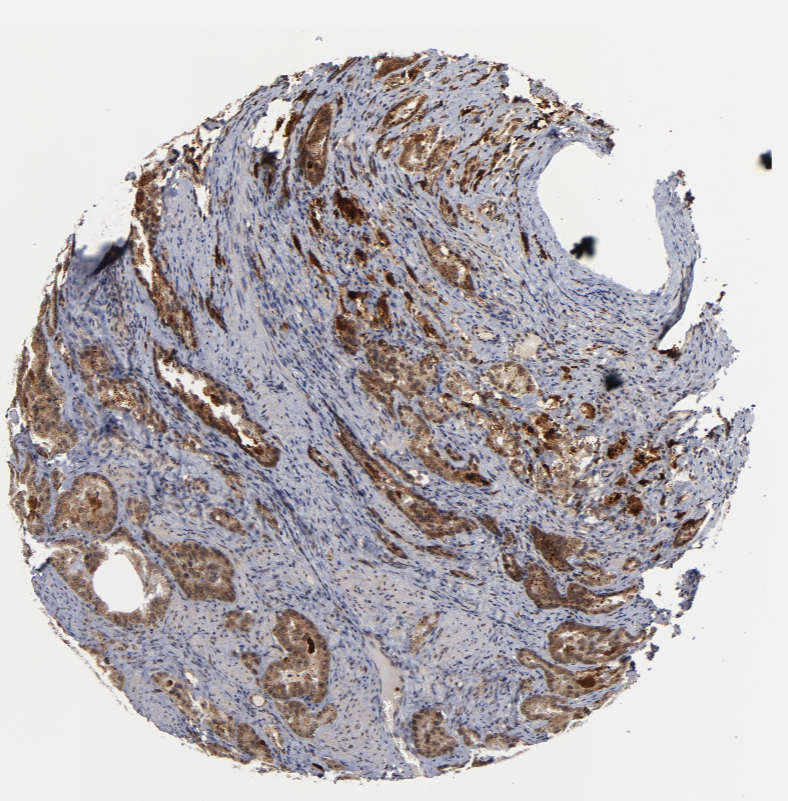


HPA003274 prostate normal medium


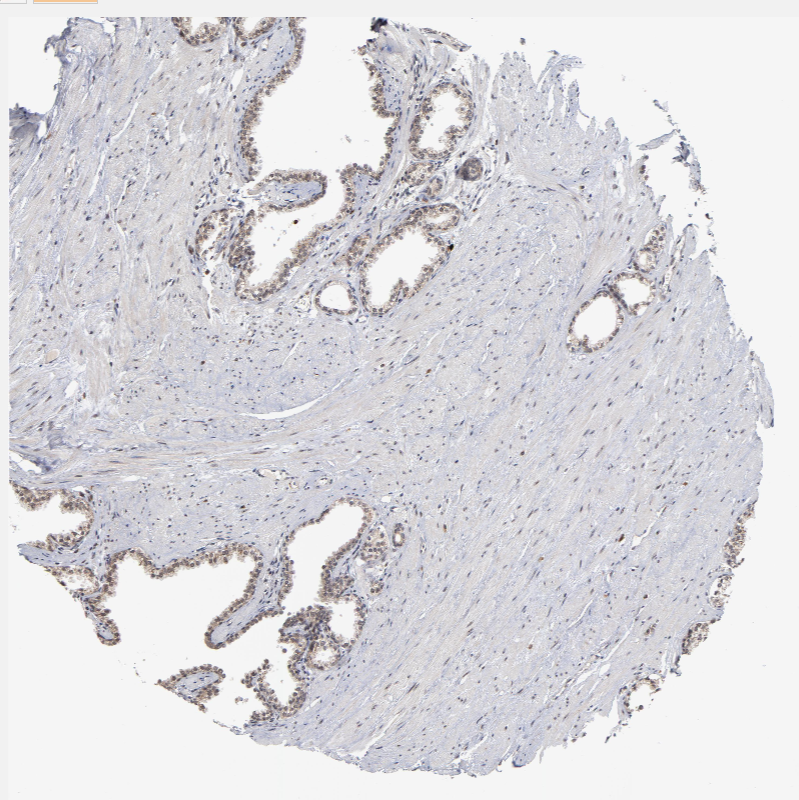


**Supplementary figure 5**. The pan-cancer Spearman analysis of clinical phenotype and ZNF419 expression.


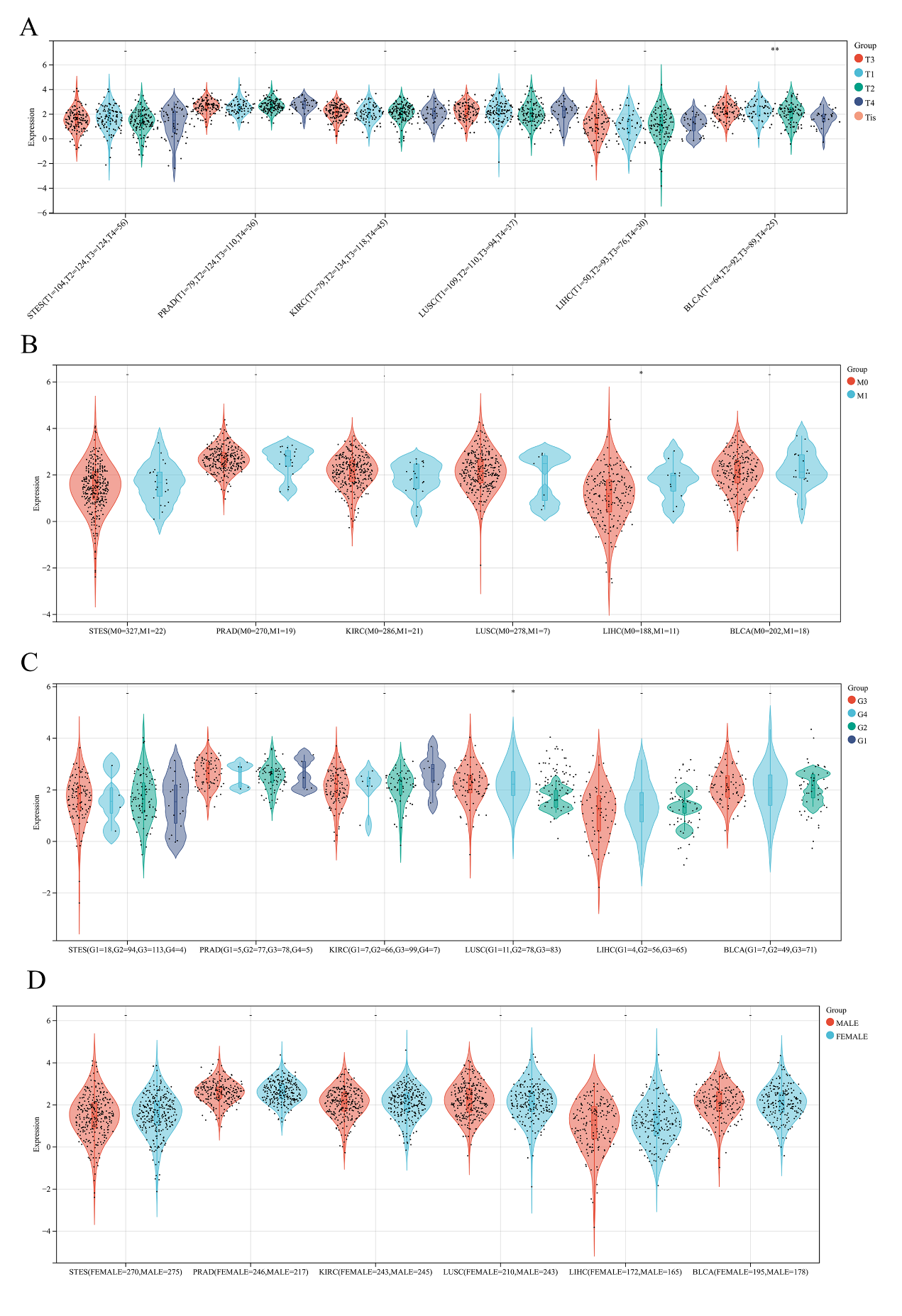


(A) the correlation of ZNF419 expression with T stage; (B) the correlation of ZNF419 expression with M stage; (C) the correlation of ZNF419 expression with cancer grade; (D) the correlation of ZNF419 expression and patient gender.
